# Sexually Dimorphic Regulation of MiR‐29a/c‐3p in Human Endothelial Cells: Cell Functions and Transcriptome

**DOI:** 10.1002/jcp.70199

**Published:** 2026-06-14

**Authors:** Si‐yan Zhang, Colman I. Freel, Chi Zhou, Jing Zheng

**Affiliations:** ^1^ Department of Obstetrics and Gynecology University of Wisconsin–Madison Madison Wisconsin USA; ^2^ School of Animal and Comparative Biomedical Sciences University of Arizona Tucson Arizona USA

**Keywords:** angiogenic factors, endothelial function, microRNAs, sexual dimorphism, transcriptome

## Abstract

Preeclampsia (PE) is a major cause of maternal and fetal morbidity and mortality during pregnancy. PE is characterized by widespread endothelial dysfunction in mothers and fetuses. The etiology of PE remains elusive, but the dysregulation of microRNAs (miRNAs) in endothelial cells may contribute to the pathogenesis of PE. We have reported that PE downregulates expression of two miRNAs, miR‐29a‐3p and miR‐29c‐3p (miR‐29a/c‐3p), and knockdown of miR‐29a/c‐3p impairs functions of human umbilical vein endothelial cells (HUVECs). Herein, we tested the hypothesis that knockdown of miR‐29a/c‐3p sex‐specifically impairs cellular responses to vascular endothelial growth factor‐A (VEGFA) and fibroblast growth factor 2 (FGF2) via disrupting the transcriptome in HUVECs. MiR‐29a/c‐3p were knocked down using miR‐29c‐3p inhibitors in male and female HUVECs. Chemotactic and proliferative responses to VEGFA and FGF2 were assessed. RNA‐seq was performed to identify miR‐29a/c‐3p regulated genes and pathways. Knockdown analysis demonstrated that miR‐29c‐3p inhibitors decreased miR‐29a/c‐3p levels by over 95% in male and female HUVECs. Functionally, miR‐29c‐3p inhibitors suppressed VEGFA‐, but not FGF2‐stimulated chemotaxis by 26% in male, but not female HUVECs. RNA‐seq revealed that miR‐29a/c‐3p inhibitors dysregulated 47 and 118 genes in male and female HUVECs, respectively. Bioinformatics analyses showed that miR‐29a/c‐3p‐regulated genes were differently associated with hypertension, heart, angiogenesis, and immunology in male and female HUVECs. These data demonstrate that knockdown of miR‐29a/c‐3p sex‐specifically affects cellular responses to VEGFA and FGF2 in HUVECs, possibly via disrupting the transcriptome and relevant pathways. These miR‐29a/c‐3p‐regulated genes might represent promising sex‐specific therapeutic targets for PE‐associated endothelial dysfunction pending further in vivo and clinical verification.

## Graphical abstract

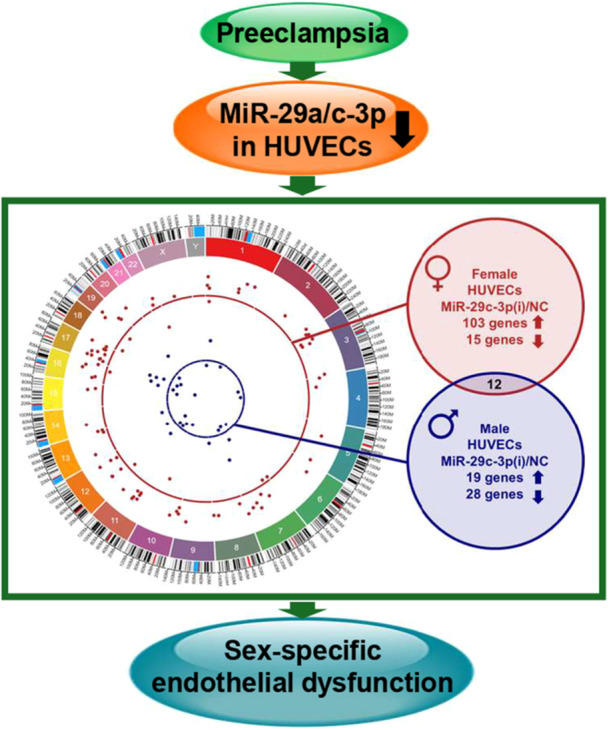

## 1 Introduction

Preeclampsia (PE) is a human pregnancy‐specific hypertensive disorder, which is characterized by widespread vascular dysfunction (Boeldt and Bird 2017; Phipps et al. 2019). PE affects 2%−8% of human pregnancies and accounts for over 50,000 maternal deaths worldwide each year (Duley 2009; Phipps et al. 2019). Women with PE and their offspring also have increased risks of developing cardiovascular diseases later in life (Bellamy et al. 2007; Kajantie et al. 2009; Staley et al. 2015). Thus, a better understanding of the mechanisms underlying vascular dysfunction associated with PE will help us more effectively combat PE.

Endothelial dysfunction contributes to PE‐related maternal vascular dysfunction (e.g., hypertension, proteinuria, and edema) (Roberts and Cooper 2001; Tomimatsu et al. 2019) and increased risks of developing future cardiovascular diseases (Chambers 2001; Powe et al. 2011). PE also induces fetal endothelial and vascular dysfunction (Boeldt and Bird 2017; Wang et al. 2002; Zhou et al. 2020). For example, human umbilical vein endothelial cells (HUVECs) from PE pregnancy exhibit reduced expression of endothelial nitric oxide (NO) synthase, weakened monolayer integrity, and impaired Ca^2+^ signaling (Boeldt and Bird 2017; Wang et al. 2002; Zhou et al. 2020), and impaired cellular responses to growth factors and cytokines (Zhou et al. 2023; Zhou et al. 2019). Thus, eluciating the mechanisms underlying PE‐associated endothelial dysfunction is critical for developing preventive and therapeutic strategies for PE.

Emerging evidence has suggested that dysregulation of functional RNAs, including microRNAs (miRNAs), contributes to the mechanisms governing PE‐associated endothelial dysfunction (Chen and Wang 2013; Munjas et al. 2021; Zhou et al. 2019), which could be mediated via a sex‐specific manner (Zhou et al. 2023; Zhou et al. 2019). Many miRNAs are differentially expressed in various tissues (e.g., placentas and circulating endothelial cells) from PE patients (Enquobahrie et al. 2011; Lv et al. 2019). We have also reported that PE suppresses the expression of two members of the miRNA‐29 family, miRNA‐29a‐3p and miRNA‐29c‐3p (termed miR‐29a/c‐3p) in HUVECs (Zhou et al. 2017). MiR‐29a/c‐3p are known to regulate endothelial cell cycle, proliferation, tube formation, cell migration, and monolayer integrity in HUVECs in vitro (Cao et al. 2024; Yang et al. 2013; Zhou et al. 2023; Zhou et al. 2017), implying their important roles in endothelial cells. Furthermore, we have predicted that PE dysregulates miR‐29a/c‐3p target genes in a cell sex‐specific manner and reported that knockdown of miR‐29a/c‐3p leads to differential cellular responses to tumor necrosis factor‐alpha (TNF‐α) and transforming growth factor beta‐1 (TGFβ1) in male and female HUVECs, even though miR‐29a/c‐3p are similarly downregulated in female and male HUVECs (Zhou et al. 2023). These findings highlight the importance of sex‐specific regulation of miR‐29a/c‐3p in human endothelial cells.

Vascular endothelial growth factor‐A (VEGFA) and fibroblast growth factor 2 (FGF2) are two growth factors that play vital roles in regulating endothelial functions (Chen and Zheng 2014; Wang and Zheng 2012). Our previous report has demonstrated that knockdown of miR‐29a/c‐3p suppresses VEGFA‐ and FGF2‐induced cell mobility (assayed using a wound healing assay), while they do not significantly affect cell proliferation and monolayer integrity in HUVECs (Zhou et al. 2017). However, it is unclear whether downregulation of miR‐29a/c‐3p sex‐specifically impairs cell chemotaxis and proliferation responses to VEGFA and FGF2 via disrupting the transcriptome in HUVECs. Therefore, this study aimed to further investigate the sexually dimorphic roles of miR‐29a/c‐3p in VEGFA‐ and FGF2‐induced endothelial functions in HUVECs, potentially providing sex‐specific therapeutic strategies for PE‐associated endothelial and vascular dysfunction.

## 2 Materials and Methods

### 2.1 Ethical Approval

All procedures for human tissue collection were conducted in accordance with the Declaration of Helsinki. All subjects gave written, informed consent. The umbilical cord collection was approved by the Institutional Review Board of UnityPoint Health‐Meriter Hospital (Madison, WI) and the Health Sciences Institutional Review Boards of the University of Wisconsin‐Madison (Protocol number 2004‐006). Patients aged 18–40 years with a normotensive singleton pregnancy were recruited by the research staff in the University of Wisconsin Department of Obstetrics and Gynecology. Exclusions included fetuses with known chromosomal abnormalities or fetal growth restriction, and mothers with pre‐rupture of membranes, smokers, and other severe disorders (e.g., diabetes, autoimmune disorders, chronic hypertension, and heart and liver diseases).

### 2.2 Isolation, Purification, and Characterization of HUVECs

HUVECs were immediately isolated from human umbilical cords after delivery at term (Table 1), as described (Zhao et al. 2025; Zhou et al. 2023; Zhou et al. 2019; Zhou et al. 2017). After isolation, endothelial cell preparations were purified using CD31 Dynabeads (catalog # 11155D, Invitrogen, Carlsbad, CA, USA) and cultured in endothelial cell media (ECM) at 37°C in 5% CO_2_% and 95% air. This ECM consists of ECMb media (catalog # 1001‐b, ScienCell, Carlsbad, CA, USA) supplemented with 2% fetal bovine serum, 1% penicillin/streptomycin, 37.5 mg/L endothelial cell growth supplement (catalog # 1052, ScienCell), and 1% amphotericin B (catalog # 15290018, Thermo Fisher Scientific, Waltham, MA). Endothelial phenotypes were further verified by their cobblestone morphologies and contact inhibition characterization at confluence. Primary HUVEC preparations at passage 2 or 3 were randomly selected and used in this study.

**Table 1:** Patient demographics for HUVECs used in cell functions, RNA‐seq, and RT‐qPCR.

	Cell sex	*n*	Maternal BMI	Maternal age (years)	Gestational age (weeks)	Fetal weight (g)	Systolic BP (mmHg)	Diastolic BP (mmHg)
VEGFA‐chemotaxis	Male	7	24.5 ± 4.9	32.6 ± 3.7	39.4 ± 0.7	3727.1 ± 315.3	118.4 ± 9.4	76.0 ± 8.7
	Female	6	23.0 ± 1.7	32.8 ± 4.8	39.8 ± 0.8	3607.5 ± 377.0	118.5 ± 12.5	64.7 ± 12.1
	*p* value		> 0.05	> 0.05	> 0.05	> 0.05	> 0.05	> 0.05
FGF2‐chemotaxis	Male	5	22.9 ± 2.9	33.5 ± 1.9	39.5 ± 0.8	3606.0 ± 387.9	113.6 ± 10.0	76.6 ± 8.6
	Female	5	23.5 ± 1.5	33.8 ± 4.7	39.7 ± 0.9	3669.0 ± 386.4	122.4 ± 9.0	64.8 ± 14.2
	*p* value		> 0.05	> 0.05	> 0.05	> 0.05	> 0.05	> 0.05
Cell proliferation	Male	5	23.3 ± 3.7	31.0 ± 2.4	39.7 ± 0.8	3518.0 ± 250.8	113.2 ± 9.4	76.2 ± 7.7
	Female	5	24.2 ± 1.5	33.4 ± 4.5	39.3 ± 0.6	3494.0 ± 526.8	111.2 ± 11.0	71.4 ± 7.9
	*p* value		> 0.05	> 0.05	> 0.05	> 0.05	> 0.05	> 0.05
RNA‐seq	Male	3	21.6 ± 0.8	33.3 ± 3.8	39.6 ± 0.5	3616.7 ± 448.4	118.7 ± 8.1	73.3 ± 5.5
	Female	4	23.3 ± 2.8	32.3 ± 4.8	39.2 ± 1.9	3272.3 ± 41.8	119.5 ± 14.1	75.8 ± 9.5
	*p* value		> 0.05	> 0.05	> 0.05	> 0.05	> 0.05	> 0.05
RT‐qPCR	Male	8	24.9 ± 2.9	32.1 ± 3.2	39.3 ± 0.4	3693.8 ± 404.6	117.4 ± 10.2	76.4 ± 6.0
	Female	8	24.1 ± 2.3	33.9 ± 3.6	39.1 ± 1.2	3413.1 ± 328.2	118.4 ± 10.7	71.4 ± 13.3
	*p* value		> 0.05	> 0.05	> 0.05	> 0.05	> 0.05	> 0.05

*Note:* Exclusions included fetuses with known chromosomal abnormalities or fetal growth restriction, and mothers with pre‐rupture of membranes, smokers, and other severe disorders (e.g., diabetes, autoimmune disorders, chronic hypertension, and heart and liver diseases). All data are presented as means ± SD. Data were analyzed using the Mann−Whitney Rank Sum Test or Student's *T* test. All patients are Caucasians and without a current or history of major complications.

Abbreviations: BMI, body mass index; BP, blood pressure; RNA‐seq, RNA‐sequencing; RT‐qPCR, reverse transcription‐quantitative PCR.

To eliminate potential mycoplasma contamination, cell preparations were treated with 0.1% mycoplasma elimination cocktails (MycoAway 1000X Mycoplasma Cocktail, catalog # G398, Applied Biological Materials, Richmond, BC, Canada) in ECM for 2 weeks following the manufacturer's instructions. The elimination of mycoplasma was confirmed using a PCR mycoplasma detection kit (catalog # G238, Applied Biological Materials). All cells used were negative for mycoplasma.

### 2.3 Knockdown of miR‐29a/c‐3p

Knockdown of miR‐29a/c‐3p in HUVECs was conducted as described (Zhou et al. 2023; Zhou et al. 2017). In the preliminary study, we observed that either 50 or 100 nM hsa‐miR‐29c‐3p miRCURY LNA miRNA Power Inhibitor (miR‐29c‐3p(i), catalog # YI04105460‐DDA, Qiagen, Valencia, CA, USA) similarly decreased miR‐29a/c‐3p by over 95% as compared with 50 or 100 nM miRCURY LNA Power Inhibitor Negative Control A (NC, catalog # YI00199006‐DDA, Qiagen) at 24, 48, and 72 h. This knockdown efficiency was comparable to the 90% reduction we previously reported using the miScript miRNA Inhibitors (Zhou et al. 2023; Zhou et al. 2017).

HUVECs seeded in 24‐well plates (120,000 cells/well) were transfected with 50 nM miR‐29c‐3p(i) or 50 nM NC in HiPerfect Transfection reagent (catalog # 301705, Qiagen) for 48 h.

### 2.4 RNA Isolation

RNA isolation was performed as described (Zhao et al. 2025; Zhao et al. 2025; Zhou et al. 2019). Sub‐confluence (70%−80%) HUVECs seeded in 24‐well plates were treated with 50 nM miR‐29c‐3p(i) or NC for 48 h. Total RNA was isolated using the RNeasy Mini kit (catalog # 74104, Qiagen). The concentration and quality of each RNA sample were assessed using a NanoDrop ND‐2000 spectrophotometer (NanoDrop Technologies, Wilmington, DE) and an Agilent 2100 bioanalyzer (Agilent Technologies, Santa Clara, CA, USA). Only RNA samples with high RNA purity and integrity number (1.9 < OD260/280 < 2.1, RNA integrity number [RIN] > 8.0) were used in this study.

### 2.5 RT‐qPCR

To verify knockdown of miR‐29a/c‐3p, RT‐qPCR was performed as described (Zhou et al. 2023; Zhou et al. 2017). Briefly, small RNA fragments enriched in total RNA isolated from each sample were reverse transcribed into cDNA using a miScript II RT Kit (catalog # 218160, Qiagen). qPCR was performed using the miScript SYBRGreen PCR Kit (catalog # 218073, Qiagen) and commercially available miRNA Primer Assays (Table S1) using a StepOnePlus qPCR system (Thermo Fisher Scientific). Data were normalized first to external control (miRTC, Qiagen), followed by normalization to the geometric mean of endogenous control miRNAs (SNORD68, SNORD95, and SNORD96A).

### 2.6 Biofunctional Assays

Chemotaxis assays were conducted using 24‐multiwell FluoroBlok transwell insert systems (8 μm pores, catalog # 351158, Corning, Corning, NY) as described (Jiang et al. 2014; Jiang et al. 2013). Briefly, cells seeded in six‐well plates (420,000/well) were treated with vehicle control (VC, transfection reagent in ECMb), NC, or miR‐29c‐3p(i) for 24 h. After 4 h of serum starvation in ECMb supplemented with 2% heat‐inactivated FBS and 1% P/S, cells were added to each insert (30,000/insert). ECMb, 10 ng/mL VEGFA (catalog # 80006‐RNAB, Sino Biological, China) or 10 ng/mL FGF2 (catalog # 10014‐HNAE, Sino Biological) was added to the bottom wells. After 16 h of incubation, calcein‐AM (catalog # C3100MP, Molecular Probes, Eugene, OR, USA) was added to the bottom wells with a final concentration of 2 μg/mL and incubated for 30 min at 37°C. Migrated cells were assessed in four randomly selected fields per well using an inverted microscope (Nikon, Melville, NY, USA) equipped with a CCD camera. Cell numbers were quantified with Metamorph image analysis software (Molecular Devices, San Jose, CA, USA).

Cell proliferation was assessed using the CCK‐8 kit (catalog# CK04, Dojindo Molecular Technologies, Rockville, MD) as described (Zhou et al. 2019). Cells seeded in 96‐well plates (5000 cells/well) were treated with VC, NC, or miR‐29c‐3p(i) for 24 h. After 8 h of serum starvation in ECMb with 1% P/S, cells were treated with ECMb (as control), 10 ng/mL VEGFA, or 10 ng/mL FGF2 for 48 h (4 wells per treatment for each cell preparation). Cells were then incubated with the CCK‐8 reagent for 1 h, and absorbance at 450 nm was measured using a Synergy HT Multi‐Detection Microplate Reader (Bio‐Tek Instruments, Winooski, VT, USA).

### 2.7 Western Blot Analysis

To determine if knockdown of miR‐29a/c‐3p differentially affected VEGFA‐induced activation of mitogen‐activated protein kinase 3/1 (also termed ERK1/2), which are key protein kinases in regulating endothelial functions (Wang and Zheng 2012), in male and female HUVECs, Western blot analysis was performed as described (Zhou et al. 2017). HUVECs were treated with VC, NC, or miR‐29c‐3p(i) for 24 h. After 16 h of serum starvation in ECMb, HUVECs were treated with ECMb or 10 ng/mL VEGFA. After 10 min of treatment, cells were lysed in lysis buffer (catalog # 78501, Thermo Fisher Scientific) supplemented with protease inhibitors (catalog # PIA32953, Thermo Fisher Scientific) and protein phosphatase inhibitors (catalog # 490684500, Sigma–Aldrich, St. Louis, MO, USA). The lysates were centrifuged, and protein concentrations of the supernatants were determined. The protein samples (20 μg) were separated on SDS‐PAGE gels and electrically transferred to polyvinylidene difluoride membranes. The membranes were immunoblotted with the antibody against phospho‐ERK1/2 (1:1000; catalog # 9101, Cell Signaling Technology, Danvers, MA, USA), total ERK1/2 (1:1000; catalog # 9102, Cell Signaling Technology), and glyceraldehyde 3‐phosphate dehydrogenase (GAPDH, 1:10,000, catalog # H00002597‐M01, Thermo Fisher Scientific).

Proteins were visualized by enhanced chemiluminescence reagents (catalog # 32106; Thermo Fisher Scientific). The immunoreactive signals of the bands were recorded using Amersham ImageQuant 800 (Cytiva, Marlborough, MA, USA) and analyzed by ImageJ software. Total ERK1/2 levels were normalized to GAPDH. Phospho‐ERK1/2 levels were normalized to total ERK1/2: GAPDH. Because VEGFA induced ERK1/2 phosphorylation similarly in male and female HUVECs, the data were pooled.

### 2.8 RNA‐Seq and Data Validation

RNA‐seq was conducted at the UW‐Madison Biotechnology Center as described (Zhao et al. 2025; Zhao et al. 2025; Zhou et al. 2019). RNA libraries were generated by Takara SMARTer Stranded RNA v2‐Pico Library Prep kit (TAKARA BIO., Kusatsu, Shiga, Japan) following the manufacturer's instructions. Briefly, 10 ng of the total RNA of each sample was fragmented with Oligos Mix at 94°C for 4 min and immediately ice‐cold for 2 min. The fragmented RNA was then reverse‐transcribed into double‐stranded complementary DNAs (cDNAs). Adapter sequences for Illumina sequencing were added via PCR1 (5 cycles). After purified cDNA with AMPure Beads, ribosomal cDNA was depleted with ZapR v2 and R‐Probes v2. Subsequently, the cDNAs were further amplified by PCR 2 with 12 cycles and purified with AMPure XP Beads to generate the final library. Library quality and concentration were assessed using the Agilent Tapestation (Agilent Technologies) and Invitrogen Qubit HS cDNA Kit 3 (Invitrogen), respectively. Each final library was about 330 bp. Each library was standardized to 2 nM. Cluster generation was performed using the NovaSeq. 6000 Reagent Kit, and sequencing was conducted on an Illumina NovaSeq. 6000 with a 2 × 150 bp read length, generating an average of 165.59 million reads per sample.

To validate RNA‐seq data, RT‐qPCR was conducted in an additional set of HUVECs preparations (*n* = 8 individual cell preparations/sex, Table 1). Total RNAs from each sample were reverse‐transcribed into cDNA using the High‐Capacity cDNA Reverse Transcription Kit (catalog # 4368814, Thermo Fisher Scientific), TaqMan Gene Expression Assays (Table S2), TaqMan Fast Advanced Master Mix (catalog # 4444557, Thermo Fisher Scientific), and a StepOnePlus qPCR system (Thermo Fisher Scientific). Eleven genes with different expression patterns (Table S2) were tested. These genes were selected based on their expression direction, fold change levels, expression levels, and relevance to endothelial functions (e.g., CXCL3 and FGF18) in female and male HUVECs. CT values over 45 are considered undetected. Data were normalized to the geometric mean of three internal housekeeping genes (GAPDH, YWHAZ, and ACTB). The normalized values were then analyzed using the $2^{-\Delta \Delta C_{T}}$.

### 2.9 Bioinformatics Analysis

The RNA‐seq data were processed using the same bioinformatics pipeline by the UW‐Madison Biotechnology Center as described (Zhao et al. 2025; Zhao et al. 2025; Zhou et al. 2019). Skewer software (v.0.2.2b) was used to trim off adapters, and Phred‐scaled quality scores are about 37, which are sufficient for RNA‐seq analysis. Then, STAR aligner (v. 2.5.3a) was used to align the trimmed reads to the GRCh38 genome. RNA‐Seq by Expectation Maximization (RSEM, v.1.2.31) software was used to obtain the read counts per sample, and EdgeR (v.3.34.0) was used to identify the differentially expressed genes (DEGs) (Benjamini‐Hochberg false discovery rate [FDR]‐adjusted *p* < 0.05). A Circos plot was generated using Circa software (OMGenomics Labs, San Francisco, CA, USA) to visualize the genomic locations of DEGs. Ingenuity Pathway Analysis (IPA; www.qiagenbioinformatics.com) was used for Gene Ontology analysis.

TargetScanHuman database (v.8.0; www.targetscan.org) was utilized to predict binding sites within the 3′ UTRs of miR‐29‐3p target genes in HUVECs and their evolutionary conservation across species (Agarwal et al. 2015; Friedman et al. 2009; McGeary et al. 2019).

### 2.10 Statistical Analysis

SigmaPlot software (Systat Software, San Jose, CA, USA) was used to analyze all the data except the RNA‐seq and IPA data. Comparisons between two groups were performed using the paired *t*‐test upon passing Shapiro−Wilk Normality test, *t*‐test, or Wilcoxon Signed Rank test. For analysis with more than two groups, one‐way or two‐way repeated measures ANOVA was performed, followed by Tukey or Holm−Sidak test for all pairwise multiple comparisons. Correlations were analyzed using linear regression analysis. Data are presented as means ± SD. Differences and correlations were considered significant when *p* < 0.05 unless stated otherwise. Benjamini‐Hochberg FDR adjustment was used for multiple comparison correction when appropriate.

## 3 Results

### 3.1 Knockdown of miR‐29a/c‐3p

**Figure 1:**
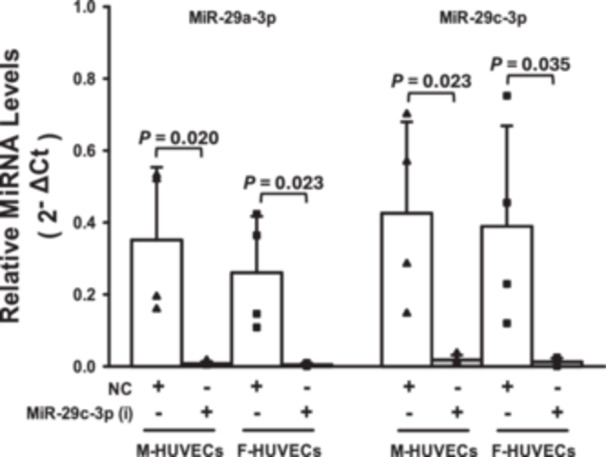
Knockdown of miR‐29a/c‐3p in male and female HUVECs. HUVECs at passage 2 were transfected with 50 nM miR‐29c‐3p (i) or NC for 48 h. Paired *t*‐test upon passing Shapiro−Wilk Normality test and two‐way repeated measures ANOVA were performed. After normalizing to the control, data are presented as means ± SD of 2^−ΔCt^. *n* = 4 cell preparations/sex. F, female; M, male; miR‐29c‐3p(i), miR‐29c‐3p inhibitor; NC, miRNA‐negative control.

RT‐qPCR analysis showed that there were no statistical differences in relative miR‐29a/c‐3p levels in NC male and female HUVECs (Figure 1). Compared with NC, miR‐29c‐3p(i) decreased miR‐29a‐3p and miR‐29c‐3p levels by over 96% in both male and female HUVECs (Figure 1), confirming the successful knockdown of miR‐29a/c‐3p in HUVECs.

### 3.2 Knockdown of miR‐29a/c‐3p Differentially Regulates Cell Functions in Male and Female HUVECs

Compared with VC, neither NC nor miR‐29c‐3p(i) alone significantly affected cell chemotaxis and proliferation in HUVECs (Figure S1), which is consistent with our previous reports (Zhou et al. 2023; Zhou et al. 2017).

**Figure 2:**
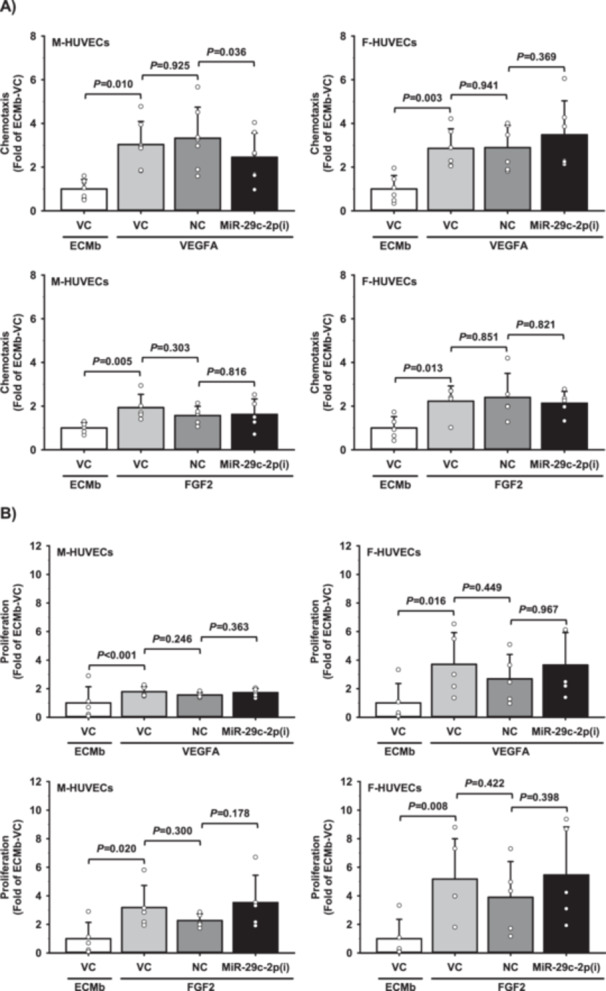
Knockdown of miR‐29a/c‐3p differentially regulates VEGFA‐ and FGF2‐induced endothelial functions in male and female HUVECs. (A) After being treated with miR‐29c‐3p(i), NC, or VC for 24 h, cells were serum‐starved for 4 h and seeded into inserts. ECMb (control), VEGFA (10 ng/mL), or FGF2 (10 ng/mL) was added to the bottom wells. After 16 h of culture, calcein AM was added to the bottom wells (final concentration of 2 μg/mL) to stain cells. Five images were taken at random sites, and migrated cells were counted. One‐way repeated measures ANOVA was performed. Data are presented as means ± SD. *n* = 5−7cell preparations/sex/group. (B) After being treated with miR‐29c‐3p(i), NC, or VC for 24 h, cells were serum‐starved for 8 h and treated with ECMb (control), VEGFA (10 ng/mL), or FGF2 (10 ng/mL) for 48 h. Cell proliferation was assessed using the CCK‐8 kit. One way repeated measures ANOVA. Data are presented as means ± SD. *n* = 5 cell preparations/sex/group. F, female; M, male; miR‐29c‐3p(i), miR‐29c‐3p inhibitor; NC, miRNA‐negative control; VC, vehicle control.

Compared with ECMb, VEGFA and FGF2 stimulated chemotaxis in VC and NC groups (Figure 2A); VEGFA in VC stimulated chemotaxis by 3.03‐ and 2.86‐fold in male and female HUVECs, respectively (Figure 2A). VEGFA in NC stimulated chemotaxis by 3.32‐ and 2.89‐fold in male and female HUVECs, respectively (Figure 2A). FGF2 in VC promoted chemotaxis by 1.93‐ and 2.23‐fold in male and female HUVECs, respectively (Figure 2A). FGF2 in NC promoted chemotaxis by 1.57‐ and 2.40‐fold in male and female HUVECs, respectively (Figure 2A). Compared to NC, miR‐29c‐3p(i) inhibited VEGFA‐stimulated chemotaxis by 26% in male, but not female, HUVECs. However, miR‐29c‐3p(i) did not significantly alter FGF2‐promoted chemotaxis in male and female HUVECs (Figure 2A).

Compared with ECMb, VEGFA and FGF2 in VC and NC stimulated cell proliferation (Figure 2B). VEGFA in VC stimulated cell proliferation by 1.78‐ and 3.71‐fold in male and female HUVECs, respectively (Figure 2B). VEGFA in NC stimulated cell proliferation by 1.55‐ and 2.69‐fold in male and female HUVECs, respectively (Figure 2B). FGF2 in VC promoted cell proliferation by 3.18‐ and 5.17‐fold in male and female HUVECs, respectively (Figure 2B). FGF2 in NC promoted cell proliferation by 2.26‐ and 3.90‐fold in male and female HUVECs, respectively (Figure 2B). However, compared with NC, miR‐29c‐3p(i) did not alter the VEGFA‐ or FGF2‐promoted proliferation in male and female HUVECs (Figure 2B).

Compared to ECMb, VEGFA elevated ERK1/2 phosphorylation by 50‐fold in HUVECs. However, compared with VC and NC, miR‐29c‐3p(i) did not alter total and phospho ERK1/2 levels in response to VEGFA in HUVECs (Figure S2).

### 3.3 Knockdown of miR‐29a/c‐3p Differentially Regulates Transcriptomes in Male and Female HUVECs

**Figure 3:**
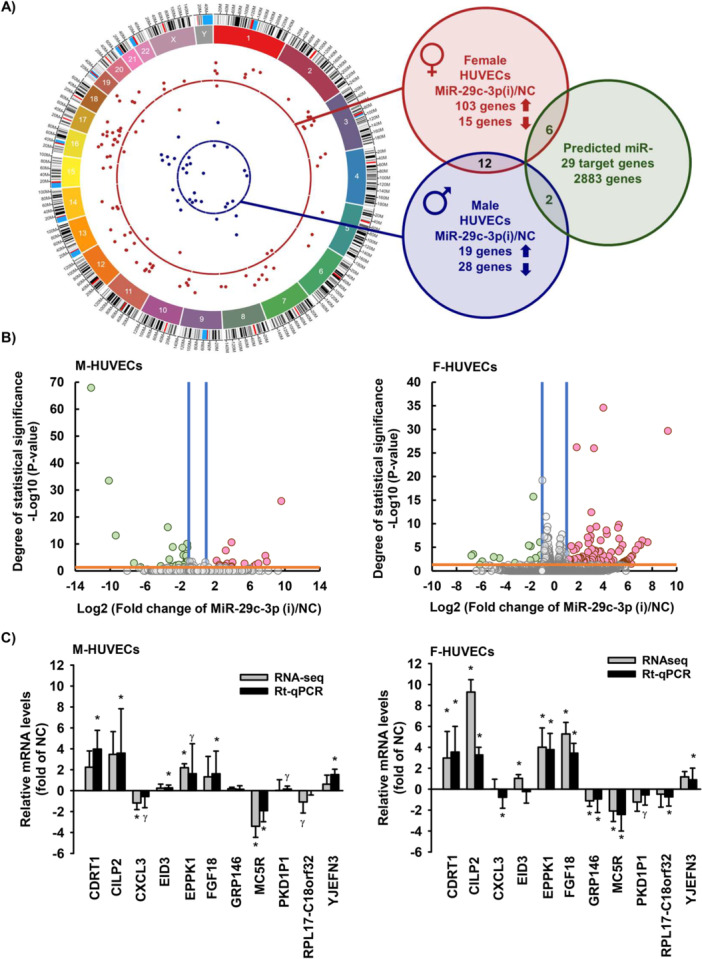
Knockdown of miR‐29a/c‐3p differentially regulates transcriptome in male and female HUVECs. (A) HUVECs at passage 2 were transfected with 50 nM miR‐29c‐3p(i) or NC for 48 h. Total RNAs were subjected to RNA‐seq analysis. Circos plot illustrates the chromosomal position of DEGs. DEGs in female (red dots) and in male (blue dots) cells. Each dot represents one gene. The numbers and letters in the outer ring indicate the chromosomal location. For each scatter plot track, dots outside and inside the centerline represent upregulated and downregulated genes, respectively. *n* = 3 and 4 for male and female, respectively. (B) Volcano plots showing DEGs in male and female HUVECs. *n* = 3 and 4 for male and female, respectively. Gray dots, no significant difference; red and green dots, >twofold up‐ and down‐regulation, respectively (FDR‐adjusted *p* < 0.05) in NC versus miR‐29c‐3p(i); Orange line, FDR *p*‐value = 0.05; F, female; M, male; miR‐29c‐3p(i), miR‐29c‐3p inhibitor; NC, miRNA‐negative control. (C) RT‐qPCR validation of RNA‐seq data in male and female HUVECs. The Mann–Whitney rank‐sum test or Student's *t* test was performed. Data are presented as means ± SD. *n* = 8 cell preparations/sex/group. *Means differ (FDR‐adjusted *p* < 0.05) from NC; ^γ^Means differ (0.1 > FDR‐adjusted *p* > 0.05) from NC.

RNA‐seq revealed that miR‐29c‐3p(i) differentially regulated the transcriptome in male and female HUVECs (Figure 3A,B; Table 2 and Table S3). In male HUVECs, miR‐29c‐3p(i) downregulated 28 genes, while upregulated 19 (Figure 3A; Table S3). In female HUVECs, miR‐29c‐3p(i) downregulated 15 genes and upregulated 103 genes (Figure 3A; Table S3). Among these DEGs identified in male and female HUVECs, 12 were common between female and male HUVECs (Table 2; Figure 3A; Table S3). Of these 12 common DEGs, 4 (MC5R, EBI3, SPX, and RRAD) were downregulated, while 8 were upregulated (LAT2, ADPRHL1, AC073957.3, LINC00565, EPPK1, AOC3, EFCAB8, and AC092139.2). One DEG (GCNA) was located on the X chromosome in female cells, while none was located on the X chromosome in male cells (Figure 2A; Table S3). No DEGs were located on the Y chromosome in male cells (Figure 3A; Table S3).

**Table 2:** MiR‐29c‐3p(i)‐commonly regulated genes in male and female HUVECs.

Gene ID	Gene symbol	Male HUVECs		Female HUVECs	
		Log2 (MiR‐29c‐3p(i)/NC)	FDR‐adjusted *P*‐Value	Log2 (MiR‐29c‐3p(i)/NC)	FDR‐adjusted *P*‐Value
ENSG00000215529	EFCAB8	6.48	1.75E‐02	5.20	7.41E‐10
ENSG00000131471	AOC3	5.38	1.91E‐03	4.27	2.58E‐09
ENSG00000272923	AC092139.2	3.40	1.95E‐03	2.94	1.26E‐02
ENSG00000260910	LINC00565	3.24	2.88E‐08	3.24	1.04E‐26
ENSG00000086730	LAT2	3.15	1.71E‐02	2.13	2.47E‐03
ENSG00000273151	AC073957.3	3.09	1.35E‐02	2.61	4.61E‐03
ENSG00000153531	ADPRHL1	2.51	6.03E‐03	2.74	8.06E‐08
ENSG00000261150	EPPK1	2.19	1.49E‐04	4.01	2.59E‐35
ENSG00000134548	SPX	−1.25	1.28E‐04	−1.93	6.32E‐06
ENSG00000166592	RRAD	−1.62	1.60E‐06	−1.12	4.20E‐02
ENSG00000105246	EBI3	−1.66	1.16E‐02	−1.96	5.89E‐03
ENSG00000176136	MC5R	−3.40	6.40E‐17	−2.09	4.11E‐06

*Note:* HUVECs were treated with 50 nM miR‐29‐c‐3p(i) or NC for 48 h. *p* < 0.05 is considered significantly differentially expressed. *n* = 4 and 3 for F‐ and M‐HUVECs preparations.

Abbreviations: FDR‐adjusted *p* value: Benjamini Hochberg adjusted *p* value; MiR‐29c‐3p(i), miR‐29c‐3p inhibitor; NC, negative control.

Only 8 DEGs identified in the current study overlapped with a list of 2,883 miR‐29a/c‐3p target genes that we previously predicted based on the TarBase v.8 and microT‐CDS databases (Zhou et al. 2023). Among these 8 DEGs, six were modulated in female HUVECs (upregulated: FGF18, PALM2‐AKAP2, SELPLG, CILP2, and AC093525.2; downregulated: AQP1), while 2 were downregulated in male HUVECs (TNFRSF9 and CLDN1) (Figure 3A; Table S3).

TargetScanHuman (v8.0) predicted broadly conserved miR‐29a/c‐3p binding sites within the 3′ UTRs of three target genes in female (PALM2‐AKAP2, CILP2, and TMEM256‐PLSCR3) and one in male (CLDN1) HUVECs. These sites are highly conserved across multiple vertebrate species, including chimpanzee, mouse, rat, pig, and brown bat (Table S7).

RT‐qPCR validation on 11 selected genes (Figure 3C) showed a strong correlation between the RT‐qPCR and RNA‐seq data (*r* = 0.928 and 0.863 for male and female cells, respectively, Figure S3), indicating the overall reliability of the RNA‐seq data.

### 3.4 Knockdown of miR‐29a/c‐3p Differentially Regulates Cardiovascular Health‐, Immune System Function‐ and Endothelial Function‐Associated Genes in Male and Female HUVECs

**Figure 4:**
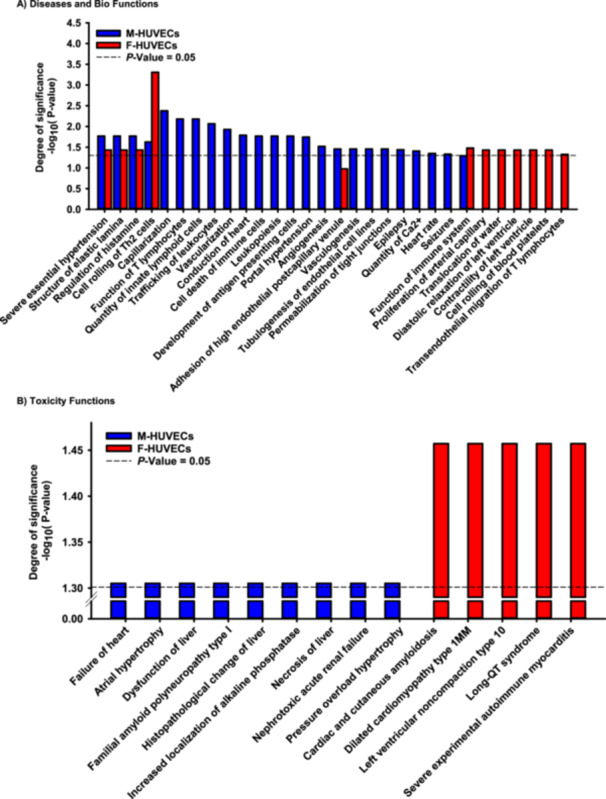
Knockdown of miR‐29a/c‐3p differentially regulates pathways in male and female HUVECs. DEGs induced by miR‐29c‐3p(i) were subjected to IPA analysis. (A) Diseases and Bio Functions and (B) Toxicity Functions. Significant enrichment was determined using IPA software (*p* < 0.05, Fisher's exact test). Dotted line: *p* = 0.05. *n* = 3 and 4 for male and female, respectively. F, female; M, male.

IPA predicted that the DEGs induced by miR‐29c‐3p(i) were enriched in many diseases and biological functions (Table S4) and toxicity functions (functions associated with damage effect to the liver, kidney, and heart muscle) (Table S5). The selected ones, which are highly relevant to cardiovascular functions, immune system function, or endothelial functions, are present in Figure 4.

The miR‐29c‐3p(i)‐induced DEGs were significantly enriched in diseases and biological functions in HUVECs (Figure 4A; Table S4). Among these functions, many were either common or unique in male and female HUVECs. For example, severe essential hypertension, structure of elastic lamina, and cell rolling of Th2 cells were commonly enriched in male and female HUVECs; vascularization, conduction of heart, leukopoiesis, angiogenesis, and cell death of immune cells were uniquely enriched in male HUVECs; Function of the immune system, proliferation of arterial capillary, and translocation of water were solely enriched in female HUVECs.

The miR‐29c‐3p(i)‐induced DEGs in HUVECs were also enriched in toxicity functions (Figure 4B; Table S5). These toxicity functions included ones unique to male HUVECs (e.g., failure of heart, atrial hypertrophy, dysfunction of liver, and nephrotoxic acute renal failure), and unique ones to female HUVECs (e.g., long‐QT syndrome, dilated cardiomyopathy type 1MM, and severe experimental autoimmune myocarditis).

Upstream regulator analysis of the miR‐29c‐3p(i)‐induced DEGs in HUVECs predicted 8 enriched gene networks (Table S6) in male HUVECs, including TNF, IL1B, and NFkB‐regulated genes. No gene network was predicted in female HUVECs.

## 4 Discussion

In this study, we have demonstrated that knockdown of miR‐29a/c‐3p inhibits VEGFA‐stimulated chemotaxis in male HUVECs without affecting VEGFA‐ and FGF2‐induced cell proliferation. We have also demonstrated that knockdown of miR‐29a/c‐3p dysregulates the transcriptome in HUVECs in a cell‐sex‐dependent manner. Furthermore, we have predicted that miR‐29a/c‐3p target genes are tightly associated with cardiovascular health, immune system function, and endothelial functions in HUVECs. These data suggest that miR‐29a/c‐3p sex‐specifically regulate endothelial functions via altering the transcriptome in human fetal endothelial cells. These sex‐specific miR‐29a/c‐3p target genes and their function pathways may contribute to the development of PE‐associated vascular dysfunction.

We reported that knockdown of miR‐29a/c‐3p inhibits cell mobility (wound healing assay), but not cell proliferation (MTT assay) in response to VEGFA and FGF2 in HUVECs, irrespective of cell sex (Zhou et al. 2017). In the current study, we have further demonstrated that knockdown of miR‐29a/c‐3p selectively inhibited VEGFA‐, but not FGF2‐stimulated chemotaxis, exclusively in male HUVECs. This fetal sex‐specific regulation in VEGFA‐stimulated chemotaxis is consistent with our previous observation of the sex‐specific role of miR29‐a/c‐3p in cellular responses to TNF‐α and TGFβ1 in HUVECs, even though miR29‐a/c‐3p are similarly downregulated in both male and female HUVECs (Zhou et al. 2023). These findings indicate the importance of peptide factor‐ and cell sex‐specific regulation of miR‐29a/c‐3p in endothelial cells, as we suggested (Zhou et al. 2023; Zhou et al. 2017). In addition, we have confirmed that miR‐29a/c‐3p do not participate in VEGFA‐ and FGF2‐stimulated cell proliferation in male and female HUVECs, as we reported (Zhou et al. 2017). This observation is contradictory to the finding that knockdown of miR‐29a suppressed proliferation of HUVECs induced by a serum‐containing growth medium, in which a commercially purchased cell preparation without detailed information on cell origin (e.g., cell sex and passage) was used (Yang et al. 2013). These discrepancies likely resulted from differences in cell preparation and stimuli used.

The precise mechanisms that drive sexually dimorphic regulation of these endothelial responses to VEGFA are unclear. Differential activation of ERK1/2 does not appear to play a major role, as we observed in the current study and previous report (Zhou et al. 2017). It is more likely that sex‐specific regulation of the transcriptome in endothelial cells may contribute to these different cell responses, as suggested (Zhou et al. 2023; Zhou et al. 2019). Indeed, in the current study, our data demonstrate a clear divergence in gene expression between male and female HUVECs upon miR‐29a/c‐3p knockdown. We have identified a fetal sex‐specific set of miR‐29a/c‐3p target genes in HUVECs and found that these target genes are closely related to endothelial functions. Based on these transcriptomic shifts and subsequent predictive pathway analyses, we would speculate that the integrated actions of this set of DEGs are a key driver of the sex‐specific regulation of miR‐29a/c‐3p in endothelial functions.

In the current study, we have uncovered a set of miR29a/c‐3p target genes specifically in primary HUVECs since only 5.2% (8 out of 153 DEGs across male and female cells) of DEGs identified in HUVECs overlap with the predicted miR‐29 target genes (Zhou et al. 2023). One of the obvious reasons for this small overlap is that these DEGs are experimentally identified in a single population of cells and are specific to HUVECs, whereas the predicted target genes are derived from a combination of computational and experimental approaches using diverse biological data sources (Karagkouni et al. 2018; Paraskevopoulou et al. 2013). Thus, our current data underscore the importance of experimentally identifying miRNA target genes in a specific cell type, which is essential for accurately defining the transcriptomic regulation by miRNAs within that cell type.

Our observation that female HUVECs have more miR‐29a/c‐3p target genes than their male counterpart (118 vs 47 DEGs, 2.5‐fold) (Table S3) aligns with the sex‐biased trend of our previous report that PE dysregulates 4.5‐fold more miR‐29a/c‐3p target genes in female than male HUVECs (Zhou et al. 2023). These data also concur with the proposition that female HUVECs exhibit greater transcriptional susceptibility in response to PE (Zhou et al. 2019) and endogenous aryl hydrocarbon receptor ligands (Zhao et al. 2025). Moreover, among the 118 DEGs identified in female HUVECs, many are critical to endothelial functions. For instance, aquaporin‐1 (AQP1, Table S3) is a key factor that facilitates NO transportation across the endothelial membrane (Herrera et al. 2006) and promotes cell proliferation, migration, and tubule formation in HUVECs and human retinal vascular endothelial cells (Cyr et al. 2020; Herrera et al. 2006; Kaneko et al. 2008). Given that NO is critical to mediating endothelial growth and vasodilation (Wang and Zheng 2012), the downregulation of AQP1 in female HUVECs likely decreases NO transport, reducing its bioavailability, which ultimately impairs vascular functions. Unsurprisingly, genes related to endothelial functions were also found among the 47 DEGs identified in male HUVECs. For instance, CLDN1 (claudin‐1, Table S3), a tight junction protein that maintains endothelial and epithelial barrier function, is downregulated in miR‐29a/c‐3p‐knockdown HUVECs. It has been reported that downregulation of CLDN1 increased endothelial permeability in human cardiac microvascular endothelial cells (Nepali et al. 2025). This suggests that miR‐29a/c‐3p differentially regulates endothelial function–related genes in female and male HUVECs, contributing to sex‐specific regulation of vascular phenotypes.

There are 12 common DEGs (e.g., melanocortin‐5 Receptor [MC5R], Epstein‐Barr 8virus‐induced gene 3 [EBI3], Spexin [SPX], Ras Related Glycolysis Inhibitor And Calcium Channel Regulator [RRAD], Linker For Activation Of T Cells Family Member 2 [LAT2], Epiplakin 1 [EPPK1] and Amine Oxidase Copper Containing 3 [OC3]) identified in female and male HUVECs (Table S3), implying the importance of these DEGs in mediating the functions of miR‐29a/c‐3p in endothelial cells. Indeed, activation of MC5R, a G‐protein‐coupled receptor gene (Ji et al. 2022), has been shown to block high‐glucose‐suppressed angiogenesis in HUVECs (Trotta et al. 2024). In addition, RRAD (Table S3), which is downregulated in both female and male HUVECs, is related to GTP‐binding activity and calcium channel regulator activity (Yada et al. 2007). As calcium signaling is critical for endothelial functions, such as maintaining cell integrity and controlling permeability (Clapham 2007) RRAD downregulation may contribute to endothelial dysfunction. In cancer cells, RRAD is also known to influence cell proliferation, motility, and cell cycle progression (Sun et al. 2023). Further studies are needed to determine whether RRAD plays similar roles in HUVECs. Furthermore, AOC3 (or vascular adhesion protein‐1, Table S3) is an endothelial adhesion molecule that enables leukocytes to bind to the endothelial cell surface and then migrate through the vessel wall (extravasation), regulating inflammatory responses (Dunkel et al. 2014; Filip et al. 2022), Thus, the upregulation of AOC3 in both female and male HUVECs suggests miR‐29a/c‐3p may actively participate in inflammation via AOC3.

Among DEGs identified in the current study, only 1 female DEG is located on the X chromosome, with no DEG located on the Y chromosome. This suggests that sex chromosome genes may not be the primary contributors to the transcriptomic differences induced by knockdown of miR‐29a/c‐3p between female and male HUVECs. This is not surprising since similar observations have been reported that sex‐specific transcriptomic changes are induced by PE or endogenous aryl hydrocarbon receptor ligands (Zhao et al. 2025; Zhao et al. 2025; Zhou et al. 2019).

It is noted that many miR‐29a/c‐3p associated diseases and biological functions are commonly enriched in cardiovascular functions, including severe essential hypertension and the structure of the elastic lamina, in female and male HUVECs. These enriched disease and biological functions are highly related to PE (Boeldt and Bird 2017; Germain et al. 2007; Granger et al. 2001). However, given the fact that the sexually dimorphic regulation of transcriptome exists between female and male HUVECs, it is expected that the miR‐29a/c‐3p associated diseases and biological functions exhibit sex‐specific patterns. For example, the transendothelial migration of T lymphocytes and translocation of water are enriched only in female HUVECs. Interestingly, angiogenesis, tubulogenesis of endothelial cell lines, and vasculogenesis are enriched only in male HUVECs, which is in line with our observation of a male‐specific regulation of miR29‐a/c‐3p in VEGFA‐stimulated cell chemotaxis in HUVECs (Figure 2A). In addition, our current finding that the downregulation of miR‐29a/c‐3p in male HUVECs included seizures among the IPA‐predicted disease associations (Table S4) reinforces the notion that PE‐dysregulated biological processes in HUVECs are strongly associated with seizures (Zhou et al. 2019). Together, these data suggest that miR‐29a/c‐3p may influence seizure‐related pathways in PE with male offspring. Furthermore, many miR‐29a/c‐3p target biological functions and pathways are related to immune responses, including cell rolling of Th2 cells, regulation of histamine in female and male HUVECs, transendothelial migration of T lymphocytes in female HUVECs, function of T lymphoid cells, quantity of innate lymphoid cells, trafficking of leukocytes, and cell death of immune cells in male HUVECs. This supports the notion that the miR‐29 family participates in regulating immune cell function (Deng et al. 2021; Fabbri et al. 2007; Pathania et al. 2024).

Several limitations inherent to this study should be acknowledged. First, the relatively small sample size, particularly for the RNA‐seq analysis, may limit the generalizability of the findings, even though a relatively large sample size was used in RT‐qPCR validation. Additionally, the use of donor‐derived primary cells introduces potential biological heterogeneity that extends beyond sex differences. Second, our study relies exclusively on an in vitro HUVEC model, which may not fully recapitulate the complexity of in vivo endothelial biology in PE. Consequently, the lack of validation in independent cohorts or clinical samples currently limits the translational relevance of these findings. Third, the study is primarily descriptive and does not delve deeply into the functional roles of individual miR‐29a/c‐3p targets identified. Future investigations using gene knockdown, overexpression, or pharmacological modulation could provide more mechanistic insights. Furthermore, it is important to note that the pathway analysis and the mRNA binding site of miRNA are predictive rather than experimentally validated and therefore should be interpreted with caution. Fourth, our experimental design focused on a single miRNA inhibition approach. Future investigations incorporating complementary overexpression models or target‐specific rescue experiments are necessary to strengthen causal interpretations. Finally, the underlying mechanism by which miR‐29a/c‐3p knockdown differentially affects cell migration in female and male HUVECs remains unclear and warrants further exploration.

In conclusion, this study reveals the growth factor‐ and sex‐specific regulation of miR‐29a/c‐3p function in human endothelial cells, possibly via disrupting the transcriptome. The identified miR‐29a/c‐3p target genes and associated pathways may represent potential therapeutic targets for the prevention and treatment of PE‐associated endothelial dysfunction. However, given the nature of these in vitro findings, further in vivo and clinical studies are required to validate the functional relevance of these targets in endothelial and vascular biology.

## Author Contributions

Jing Zheng conceived and designed this study. Si‐yan Zhang, Colman I. Freel, and Chi Zhou acquired and analyzed the data. Si‐yan Zhang drafted the manuscript. Colman I. Freel, Chi Zhou, and Jing Zheng revised the manuscript. All authors approved the final version of the manuscript and agreed to be accountable for all aspects of the work in ensuring that questions related to the accuracy or integrity of any part of the work are appropriately investigated and resolved. All individuals designated as authors qualify for authorship, and all those who qualify for authorship are listed.

## Conflicts of Interest

The authors declare no conflicts of interest.

## Supporting information

Supporting File 1

Supporting File 2

Supporting File 3

Supporting File 4

Supporting File 5

Supporting File 6

Supporting File 7

Supporting File 8

## Data Availability

Supporting data are available within the article and its online Supporting Information S1: Files (Tables S1–S7, Figures S1–S3) at Figshare: https://doi.org/10.6084/m9.figshare.30715988. RNA‐seq data have been deposited in the NCBI Gene Expression Omnibus database (accession number GSE310550).
