# Supplementary material for: Sexually Dimorphic Regulation of MiR‐29a/c‐3p in Human Endothelial Cells: Cell Functions and Transcriptome
Source: J Cell Physiol. 2026 Jun 14;241(6):e70199. doi: 10.1002/jcp.70199 (PMC13266284; doi:10.1002/jcp.70199)
Supplement: Supplementary file 1 — Supporting File 1 [file JCP-241-0-s006.pdf]

Fig.S1

A) B)

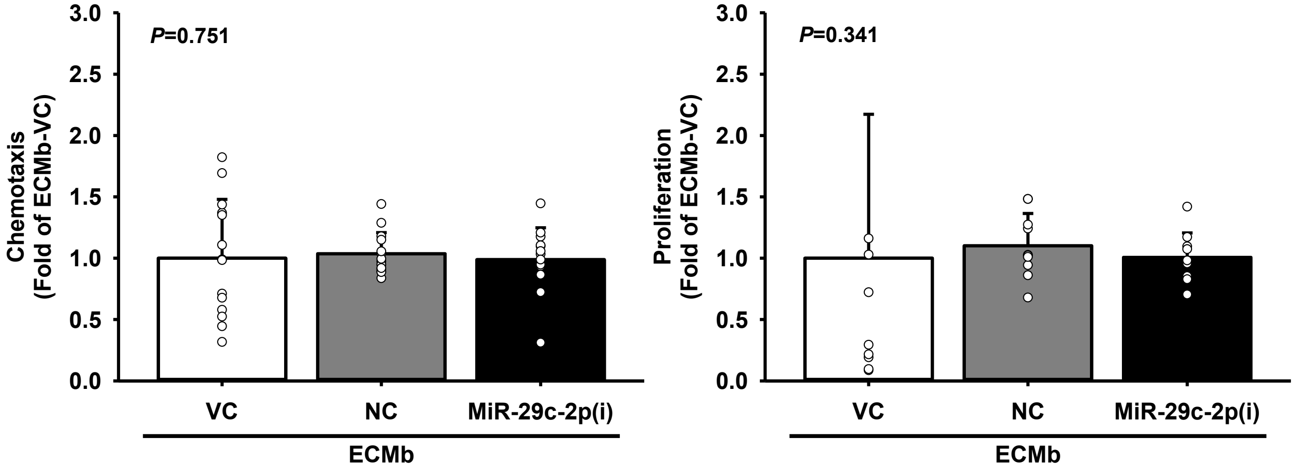

Fig. S1. Knockdown of miR-29a/c-3p doesn't significantly affect chemotaxis and cell proliferation in ECM-b in HUVECs. **A)** After being treated with miR-29c-3p(i), NC, or VC for 24 h, cells were serum-starved for 4 h and seeded into inserts. ECMb was added to the bottom wells. After 16 h of culture, calcein AM was added to the bottom wells (final concentration of 2 µg/ml) to stain cells. Five images were taken at random sites, and migrated cells were counted. One Way Repeated Measures ANOVA was performed. Data are presented as means ± SD. n =14 cell preparations/group (male:8, female:6). VC: Vehicle control; NC: miRNA-negative control; miR-29c-3p(i): miR-29c-3p inhibitor. **B)** After being treated with miR-29c-3p(i), NC, or VC for 24 h, cells were serum-starved for 8 h and treated with ECMb for 48h. Cell proliferation was assessed using the CCK-8 kit. One Way Repeated Measures ANOVA. Data are presented as means ± SD. n = 10 cell preparations/group (male:5, female:5). VC: Vehicle control; NC: miRNA-negative control; miR-29c-3p(i): miR-29c-3p inhibitor.

Fig.S2

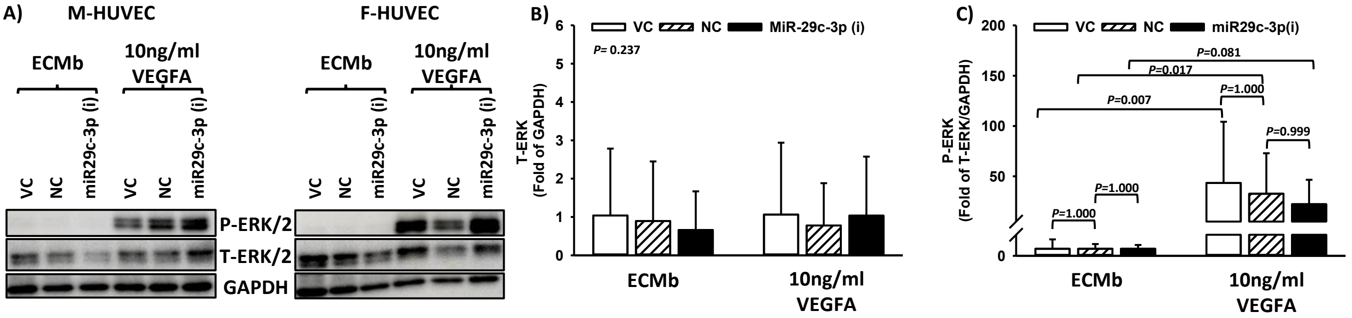

Fig. S2. Effects of miR-29a/c-3p knockdown on VEGFA-induced phosphorylation of ERK1/2 in HUVECs. After a 24-hour treatment with VC, NC, or miR-29c-3p(i), followed by a 16-hour serum starvation, HUVECs were treated with ECMb (control) or VEGFA (10 ng/mL) for 10 minutes. Protein samples were subjected to Western blotting. **A)** Representative blots in male HUVECs. **B)** Effects of miR-29a/c-3p knockdown on VEGFA-induced T-ERK1/2. **C)** Effects of miR-29a/c-3p knockdown on VEGFA-induced P-ERK1/2. Data are presented as means ± SD. n = 8 cell preparations/group (Male:4; Female:4). One way repeated measures ANOVA was performed. M:Male; F: Female; VC: Vehicle control; NC: miRNA-negative control; miR-29c-3p(i): miR-29c-3p inhibitor; P-ERK1/2: phospho-ERK1/2 ; T: total-ERK1/2.

Fig.S3

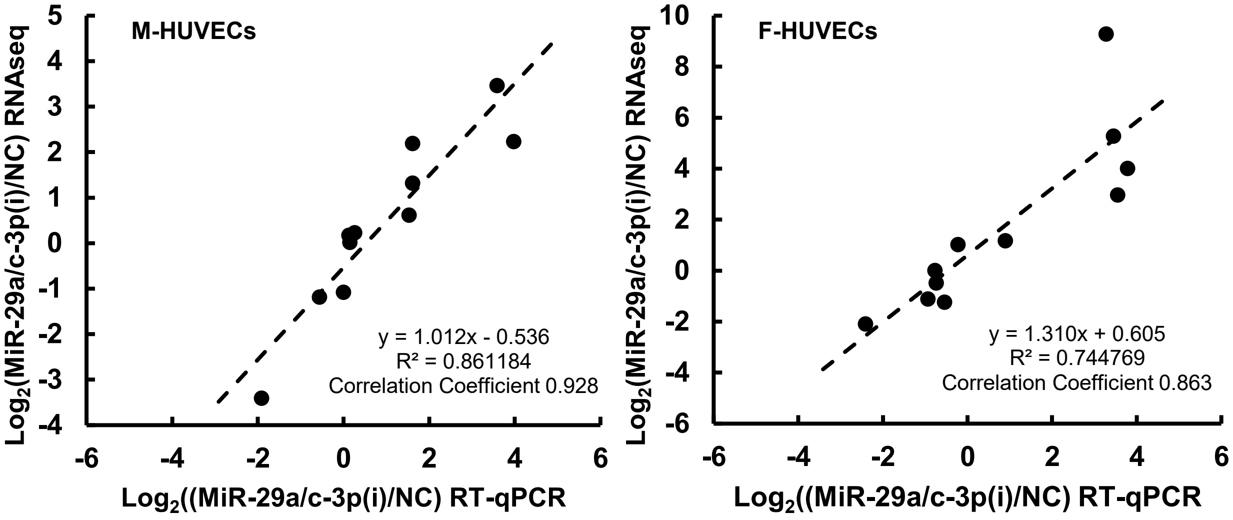

Fig. S3. Linear regression analysis comparing RNA-seq and RT-qPCR results. 11 genes identified by RNA-seq were validated using RT-qPCR. For RNA-seq, n = 3-4 individual cell preparations/ sex/group (Male = 3, Female = 4). For RT-qPCR, n = 8 individual cell preparations/ sex/group. M:Male; F: Female; NC: miRNA-negative control; miR-29c-3p(i): miR-29c-3p inhibitor.
