# Supplementary material for: Sexually Dimorphic Regulation of MiR‐29a/c‐3p in Human Endothelial Cells: Cell Functions and Transcriptome
Source: J Cell Physiol. 2026 Jun 14;241(6):e70199. doi: 10.1002/jcp.70199 (PMC13266284; doi:10.1002/jcp.70199)
Supplement: Supplementary file 2 — Supporting File 2 [file JCP-241-0-s004.docx]

| Table S1. MiRNA RT-qPCR primers. | | |
| --- | --- | --- |
| Mature miRNA ID | RT-qPCR Primer Assay Type | Catalog Number |
| Hsa-miR-29a-3p | Primer Assay for miRNA of interest | 339306(YP00204698) |
| Hsa-miR-29c-3p | Primer Assay for miRNA of interest | 339306(YP00204729) |
| MIRTC | Primer Assay for external control | MS00000001 |
| Hs_SNORD68 | Primer Assay for endogenous control (small nucleolar RNA) | MS00033712 |
| Hs_SNORD95 | Primer Assay for endogenous control (small nucleolar RNA) | MS00033726 |
| Hs_SNORD96A | Primer Assay for endogenous control (small nucleolar RNA) | MS00033733 |
| All primers were purchased from Qiagen. | | |
