# Supplementary material for: Sexually Dimorphic Regulation of MiR‐29a/c‐3p in Human Endothelial Cells: Cell Functions and Transcriptome
Source: J Cell Physiol. 2026 Jun 14;241(6):e70199. doi: 10.1002/jcp.70199 (PMC13266284; doi:10.1002/jcp.70199)
Supplement: Supplementary file 3 — Supporting File 3 [file JCP-241-0-s005.docx]

| Table S2. RT-qPCR primers. | | |
| --- | --- | --- |
| RNA ID | RT-qPCR Primer Assay Type | Assay ID |
| FBXW10B (CDRT1) | Primer Assay for mRNA of interest | Hs04984886_m1 |
| CILP2 | Primer Assay for mRNA of interest | [Hs00541922_m1](https://www.thermofisher.com/taqman-gene-expression/product/Hs00541922_m1?CID=&ICID=&subtype=) |
| CXCL3 | Primer Assay for mRNA of interest | Hs00171061_m1 |
| EID3 | Primer Assay for mRNA of interest | Hs07291013_s1 |
| EPPK1 | Primer Assay for mRNA of interest | [Hs01104050_s1](https://www.thermofisher.com/taqman-gene-expression/product/Hs01104050_s1?CID=&ICID=&subtype=) |
| FGF18 | Primer Assay for mRNA of interest | [Hs00826077_m1](https://www.thermofisher.com/taqman-gene-expression/product/Hs00826077_m1?CID=&ICID=&subtype=) |
| GPR146 | Primer Assay for mRNA of interest | Hs00298904_s1 |
| Mc5r | Primer Assay for mRNA of interest | Hs00271882_s1 |
| PKD1P1 | Primer Assay for mRNA of interest | Hs04964431_gH |
| RPL17-C18orf32 | Primer Assay for mRNA of interest | Hs07290782_m1 |
| YJEFN3 | Primer Assay for mRNA of interest | Hs07290578_m1 |
| YWHAZ | Primer Assay for internal control | Hs01122445_g1 |
| ACTB | Primer Assay for internal control | Hs01060665_g1 |
| GAPDH | Primer Assay for internal control | Hs02786624_g1 |
| All primers were purchased from Thermo Fisher Scientific. | | |
