# Supplementary material for: Sexually Dimorphic Regulation of MiR‐29a/c‐3p in Human Endothelial Cells: Cell Functions and Transcriptome
Source: J Cell Physiol. 2026 Jun 14;241(6):e70199. doi: 10.1002/jcp.70199 (PMC13266284; doi:10.1002/jcp.70199)
Supplement: Supplementary file 4 — Supporting File 4 [file JCP-241-0-s002.docx]

| Table S3. MiR-29c-3p(i)-dysregulated genes in HUVECs. | | | | | | | | |
| --- | --- | --- | --- | --- | --- | --- | --- | --- |
| Gene ID | Gene location on sex chromosome | Gene symbol | Male HUVECs | | Female HUVECs | | Comments | Match with prediction |
|  |  |  | Log2 (MiR-29c-3p(i)/NC) | FDR-adjusted *P*-Value | Log2 (MiR-29c-3p(i)/NC) | FDR-adjusted *P*-Value |  |  |
| **ENSG00000215529** |  | **EFCAB8** | **6.48** | **1.75E-02** | **5.2** | **7.41E-10** | **DE in both male and female HUVECs** |  |
| **ENSG00000131471** |  | **AOC3** | **5.38** | **1.91E-03** | **4.27** | **2.58E-09** | **DE in both male and female HUVECs** |  |
| **ENSG00000272923** |  | **AC092139.2** | **3.4** | **1.95E-03** | **2.94** | **1.26E-02** | **DE in both male and female HUVECs** |  |
| **ENSG00000260910** |  | **LINC00565** | **3.24** | **2.88E-08** | **3.24** | **1.04E-26** | **DE in both male and female HUVECs** |  |
| **ENSG00000086730** |  | **LAT2** | **3.15** | **1.71E-02** | **2.13** | **2.47E-03** | **DE in both male and female HUVECs** |  |
| **ENSG00000273151** |  | **AC073957.3** | **3.09** | **1.35E-02** | **2.61** | **4.61E-03** | **DE in both male and female HUVECs** |  |
| **ENSG00000153531** |  | **ADPRHL1** | **2.51** | **6.03E-03** | **2.74** | **8.06E-08** | **DE in both male and female HUVECs** |  |
| **ENSG00000261150** |  | **EPPK1** | **2.19** | **1.49E-04** | **4.01** | **2.59E-35** | **DE in both male and female HUVECs** |  |
| **ENSG00000134548** |  | **SPX** | **-1.25** | **1.28E-04** | **-1.93** | **6.32E-06** | **DE in both male and female HUVECs** |  |
| **ENSG00000166592** |  | **RRAD** | **-1.62** | **1.60E-06** | **-1.12** | **4.20E-02** | **DE in both male and female HUVECs** |  |
| **ENSG00000105246** |  | **EBI3** | **-1.66** | **1.16E-02** | **-1.96** | **5.89E-03** | **DE in both male and female HUVECs** |  |
| **ENSG00000176136** |  | **MC5R** | **-3.4** | **6.40E-17** | **-2.09** | **4.11E-06** | **DE in both male and female HUVECs** |  |
| ENSG00000281887 |  | GIMAP1-GIMAP5 | 9.6 | 1.28E-26 |  |  | DE only in male HUVECs |  |
| ENSG00000268083 |  | AC008982.1 | 7.94 | 3.34E-04 |  |  | DE only in male HUVECs |  |
| ENSG00000278299 |  | TBC1D3C | 7.77 | 2.53E-06 |  |  | DE only in male HUVECs |  |
| ENSG00000274944 |  | AL139260.3 | 6.97 | 1.67E-03 |  |  | DE only in male HUVECs |  |
| ENSG00000129993 |  | CBFA2T3 | 6.68 | 5.69E-03 |  |  | DE only in male HUVECs |  |
| ENSG00000260482 |  | AC008870.1 | 4.01 | 7.32E-04 |  |  | DE only in male HUVECs |  |
| ENSG00000267303 |  | AC011511.4 | 3.9 | 2.64E-11 |  |  | DE only in male HUVECs |  |
| ENSG00000157322 |  | CLEC18A | 3.89 | 2.33E-02 |  |  | DE only in male HUVECs |  |
| ENSG00000151079 |  | AC005833.1 | 3.86 | 4.81E-02 |  |  | DE only in male HUVECs |  |
| ENSG00000266086 |  | AC015813.2 | 2.75 | 1.80E-02 |  |  | DE only in male HUVECs |  |
| ENSG00000276956 |  | RN7SL769P | 2.54 | 1.40E-02 |  |  | DE only in male HUVECs |  |
| ENSG00000101210 |  | EEF1A2 | -1.1 | 2.35E-04 |  |  | DE only in male HUVECs |  |
| ENSG00000023445 |  | BIRC3 | -1.15 | 2.01E-09 |  |  | DE only in male HUVECs |  |
| ENSG00000163734 |  | CXCL3 | -1.18 | 1.09E-04 |  |  | DE only in male HUVECs |  |
| ENSG00000162772 |  | ATF3 | -1.19 | 9.01E-11 |  |  | DE only in male HUVECs |  |
| ENSG00000258017 |  | AC011603.2 | -1.3 | 2.54E-06 |  |  | DE only in male HUVECs |  |
| ENSG00000258529 |  | AP001781.3 | -1.58 | 4.21E-02 |  |  | DE only in male HUVECs |  |
| ENSG00000196329 |  | GIMAP5 | -1.62 | 5.69E-03 |  |  | DE only in male HUVECs |  |
| ENSG00000049249 |  | TNFRSF9 | -1.73 | 2.63E-09 |  |  | DE only in male HUVECs | Predicted miR29a/c-3p common target gene |
| ENSG00000163347 |  | CLDN1 | -1.79 | 1.03E-02 |  |  | DE only in male HUVECs |  |
| ENSG00000237181 |  | AC147651.3 | -2.14 | 5.69E-03 |  |  | DE only in male HUVECs |  |
| ENSG00000258924 |  | AC002094.1 | -2.15 | 4.84E-02 |  |  | DE only in male HUVECs |  |
| ENSG00000006283 |  | CACNA1G | -2.43 | 1.16E-02 |  |  | DE only in male HUVECs |  |
| ENSG00000259112 |  | NDUFC2-KCTD14 | -2.46 | 4.26E-02 |  |  | DE only in male HUVECs |  |
| ENSG00000106236 |  | NPTX2 | -2.46 | 1.03E-02 |  |  | DE only in male HUVECs |  |
| ENSG00000199415 |  | RNA5SP370 | -2.83 | 1.51E-09 |  |  | DE only in male HUVECs |  |
| ENSG00000254553 |  | AL033529.1 | -2.98 | 3.41E-02 |  |  | DE only in male HUVECs |  |
| ENSG00000220793 |  | AC087190.1 | -3.23 | 5.14E-05 |  |  | DE only in male HUVECs |  |
| ENSG00000188620 |  | HMX3 | -3.32 | 4.84E-02 |  |  | DE only in male HUVECs |  |
| ENSG00000139269 |  | INHBE | -4.21 | 1.77E-02 |  |  | DE only in male HUVECs |  |
| ENSG00000273433 |  | AC004080.6 | -6.5 | 4.26E-02 |  |  | DE only in male HUVECs |  |
| ENSG00000249319 |  | AC068533.4 | -7.28 | 1.35E-03 |  |  | DE only in male HUVECs |  |
| ENSG00000281000 |  | SNORD3D | -9.37 | 7.60E-14 |  |  | DE only in male HUVECs |  |
| ENSG00000267261 |  | AC099811.2 | -10.16 | 3.34E-34 |  |  | DE only in male HUVECs |  |
| ENSG00000280494 |  | MIR7641-2 | -12.18 | 1.25E-68 |  |  | DE only in male HUVECs |  |
| ENSG00000160161 |  | CILP2 |  |  | 9.28 | 2.24E-30 | DE only in female HUVECs | Predicted miR29a/c-3p common target gene |
| ENSG00000243207 |  | PPAN-P2RY11 |  |  | 7.63 | 8.39E-07 | DE only in female HUVECs |  |
| ENSG00000167880 |  | EVPL |  |  | 7.2 | 3.71E-07 | DE only in female HUVECs |  |
| ENSG00000182885 |  | ADGRG3 |  |  | 7.03 | 3.69E-06 | DE only in female HUVECs |  |
| ENSG00000171931 |  | FBXW10 |  |  | 6.88 | 3.69E-06 | DE only in female HUVECs |  |
| ENSG00000183160 |  | TMEM119 |  |  | 6.64 | 3.62E-05 | DE only in female HUVECs |  |
| ENSG00000106540 |  | AC004837.1 |  |  | 6.38 | 6.17E-04 | DE only in female HUVECs |  |
| ENSG00000157654 |  | PALM2-AKAP2 |  |  | 6.31 | 3.39E-02 | DE only in female HUVECs | Predicted miR29a/c-3p common target gene |
| ENSG00000255587 |  | RAB44 |  |  | 6.28 | 1.11E-03 | DE only in female HUVECs |  |
| ENSG00000157423 |  | HYDIN |  |  | 6.26 | 1.24E-03 | DE only in female HUVECs |  |
| ENSG00000175513 |  | TSGA10IP |  |  | 6.06 | 3.98E-03 | DE only in female HUVECs |  |
| ENSG00000274226 |  | TBC1D3H |  |  | 5.95 | 1.95E-02 | DE only in female HUVECs |  |
| ENSG00000273167 |  | AL359736.1 |  |  | 5.91 | 2.96E-02 | DE only in female HUVECs |  |
| ENSG00000259158 |  | ADAM20P1 |  |  | 5.88 | 7.16E-03 | DE only in female HUVECs |  |
| ENSG00000234210 |  | AC006372.3 |  |  | 5.79 | 9.54E-03 | DE only in female HUVECs |  |
| ENSG00000238109 |  | AC004893.1 |  |  | 5.74 | 1.95E-02 | DE only in female HUVECs |  |
| ENSG00000118137 |  | APOA1 |  |  | 5.69 | 1.30E-02 | DE only in female HUVECs |  |
| ENSG00000172940 |  | SLC22A13 |  |  | 5.66 | 1.45E-02 | DE only in female HUVECs |  |
| ENSG00000144671 |  | SLC22A14 |  |  | 5.64 | 2.01E-02 | DE only in female HUVECs |  |
| ENSG00000168427 |  | KLHL30 |  |  | 5.6 | 2.65E-02 | DE only in female HUVECs |  |
| ENSG00000162771 |  | FAM71A |  |  | 5.58 | 3.43E-06 | DE only in female HUVECs |  |
| ENSG00000142583 |  | SLC2A5 |  |  | 5.55 | 2.43E-02 | DE only in female HUVECs |  |
| ENSG00000128482 |  | RNF112 |  |  | 5.54 | 2.43E-02 | DE only in female HUVECs |  |
| ENSG00000162779 |  | AXDND1 |  |  | 5.53 | 3.39E-02 | DE only in female HUVECs |  |
| ENSG00000177291 |  | GJD4 |  |  | 5.47 | 4.97E-02 | DE only in female HUVECs |  |
| ENSG00000240023 |  | AL133163.1 |  |  | 5.47 | 4.87E-02 | DE only in female HUVECs |  |
| ENSG00000110076 |  | NRXN2 |  |  | 5.46 | 4.69E-02 | DE only in female HUVECs |  |
| ENSG00000134571 |  | MYBPC3 |  |  | 5.42 | 4.20E-02 | DE only in female HUVECs |  |
| ENSG00000178722 |  | C5orf64 |  |  | 5.41 | 4.31E-02 | DE only in female HUVECs |  |
| ENSG00000135519 |  | KCNH3 |  |  | 5.39 | 2.91E-05 | DE only in female HUVECs |  |
| ENSG00000131379 |  | C3orf20 |  |  | 5.38 | 4.97E-02 | DE only in female HUVECs |  |
| ENSG00000178732 |  | GP5 |  |  | 5.31 | 1.44E-07 | DE only in female HUVECs |  |
| ENSG00000156427 |  | FGF18 |  |  | 5.28 | 1.74E-10 | DE only in female HUVECs | Predicted miR29c-3p target gene only |
| ENSG00000111405 |  | ENDOU |  |  | 5.27 | 7.65E-05 | DE only in female HUVECs |  |
| ENSG00000261606 |  | AC091230.1 |  |  | 4.84 | 5.89E-03 | DE only in female HUVECs |  |
| ENSG00000144550 |  | CPNE9 |  |  | 4.65 | 9.54E-03 | DE only in female HUVECs |  |
| ENSG00000232716 |  | AC016831.3 |  |  | 4.65 | 1.09E-04 | DE only in female HUVECs |  |
| ENSG00000213892 |  | CEACAM16 |  |  | 4.53 | 1.49E-02 | DE only in female HUVECs |  |
| ENSG00000232656 |  | IDI2-AS1 |  |  | 4.46 | 8.65E-04 | DE only in female HUVECs |  |
| ENSG00000213578 |  | CPLX3 |  |  | 4.39 | 4.90E-02 | DE only in female HUVECs |  |
| ENSG00000114349 |  | GNAT1 |  |  | 4.31 | 3.51E-02 | DE only in female HUVECs |  |
| ENSG00000280213 |  | UCKL1-AS1 |  |  | 4.3 | 1.62E-08 | DE only in female HUVECs |  |
| ENSG00000259305 |  | ZHX1-C8orf76 |  |  | 4.19 | 7.50E-03 | DE only in female HUVECs |  |
| ENSG00000165917 |  | RAPSN |  |  | 3.98 | 5.13E-04 | DE only in female HUVECs |  |
| ENSG00000206952 |  | SNORA50A |  |  | 3.96 | 4.02E-02 | DE only in female HUVECs |  |
| ENSG00000225383 |  | SFTA1P |  |  | 3.86 | 3.08E-02 | DE only in female HUVECs |  |
| ENSG00000091128 |  | LAMB4 |  |  | 3.8 | 6.35E-03 | DE only in female HUVECs |  |
| ENSG00000136286 |  | MYO1G |  |  | 3.79 | 3.50E-07 | DE only in female HUVECs |  |
| ENSG00000105523 |  | FAM83E |  |  | 3.76 | 6.87E-05 | DE only in female HUVECs |  |
| ENSG00000175267 |  | VWA3A |  |  | 3.65 | 8.24E-05 | DE only in female HUVECs |  |
| ENSG00000109758 |  | HGFAC |  |  | 3.63 | 2.66E-02 | DE only in female HUVECs |  |
| ENSG00000115850 |  | LCT |  |  | 3.47 | 3.98E-03 | DE only in female HUVECs |  |
| ENSG00000159450 |  | TCHH |  |  | 3.43 | 4.39E-10 | DE only in female HUVECs |  |
| ENSG00000068976 |  | PYGM |  |  | 3.34 | 4.88E-05 | DE only in female HUVECs |  |
| ENSG00000280893 |  | AC009133.6 |  |  | 3.31 | 7.65E-05 | DE only in female HUVECs |  |
| ENSG00000257838 |  | AC106788.1 |  |  | 3.28 | 3.02E-04 | DE only in female HUVECs |  |
| ENSG00000165923 |  | AGBL2 |  |  | 3.27 | 4.99E-03 | DE only in female HUVECs |  |
| ENSG00000278000 |  | AC139100.2 |  |  | 3.26 | 2.69E-03 | DE only in female HUVECs |  |
| ENSG00000280407 |  | AC132872.4 |  |  | 3.14 | 1.30E-03 | DE only in female HUVECs |  |
| ENSG00000262481 |  | TMEM256-PLSCR3 |  |  | 3.08 | 4.14E-03 | DE only in female HUVECs |  |
| ENSG00000185133 |  | INPP5J |  |  | 3.06 | 2.49E-05 | DE only in female HUVECs |  |
| ENSG00000116254 |  | CHD5 |  |  | 3.02 | 3.95E-13 | DE only in female HUVECs |  |
| ENSG00000241322 |  | CDRT1 |  |  | 2.97 | 4.38E-08 | DE only in female HUVECs |  |
| ENSG00000007314 |  | SCN4A |  |  | 2.94 | 1.40E-09 | DE only in female HUVECs |  |
| ENSG00000110876 |  | SELPLG |  |  | 2.92 | 3.42E-07 | DE only in female HUVECs | Predicted miR29a-3p target gene only |
| ENSG00000103196 |  | CRISPLD2 |  |  | 2.79 | 1.47E-06 | DE only in female HUVECs |  |
| ENSG00000091536 |  | MYO15A |  |  | 2.78 | 2.52E-02 | DE only in female HUVECs |  |
| ENSG00000198156 |  | NPIPB6 |  |  | 2.76 | 1.07E-04 | DE only in female HUVECs |  |
| ENSG00000237399 |  | PITRM1-AS1 |  |  | 2.62 | 7.74E-03 | DE only in female HUVECs |  |
| ENSG00000187775 |  | DNAH17 |  |  | 2.61 | 2.07E-03 | DE only in female HUVECs |  |
| ENSG00000273599 |  | AL731571.1 |  |  | 2.43 | 7.30E-03 | DE only in female HUVECs |  |
| ENSG00000282218 |  | AL132671.2 |  |  | 2.42 | 2.61E-02 | DE only in female HUVECs |  |
| ENSG00000007312 |  | CD79B |  |  | 2.39 | 1.48E-02 | DE only in female HUVECs |  |
| ENSG00000143552 |  | NUP210L |  |  | 2.39 | 1.11E-02 | DE only in female HUVECs |  |
| ENSG00000182308 |  | DCAF4L1 |  |  | 2.29 | 1.38E-03 | DE only in female HUVECs |  |
| ENSG00000279266 |  | AC068860.1 |  |  | 2.29 | 9.37E-04 | DE only in female HUVECs |  |
| ENSG00000162461 |  | SLC25A34 |  |  | 2.2 | 4.72E-02 | DE only in female HUVECs |  |
| ENSG00000197847 |  | SLC22A20 |  |  | 2.17 | 3.83E-02 | DE only in female HUVECs |  |
| ENSG00000276710 |  | CSPG4P10 |  |  | 2.15 | 1.71E-03 | DE only in female HUVECs |  |
| ENSG00000003987 |  | MTMR7 |  |  | 2.14 | 4.80E-02 | DE only in female HUVECs |  |
| ENSG00000181418 |  | DDN |  |  | 2.04 | 8.15E-03 | DE only in female HUVECs |  |
| ENSG00000173727 |  | AP000769.1 |  |  | 2.03 | 2.83E-03 | DE only in female HUVECs |  |
| ENSG00000131480 |  | AOC2 |  |  | 1.99 | 3.98E-06 | DE only in female HUVECs |  |
| ENSG00000008300 |  | CELSR3 |  |  | 1.95 | 1.79E-03 | DE only in female HUVECs |  |
| ENSG00000144668 |  | ITGA9 |  |  | 1.83 | 1.38E-02 | DE only in female HUVECs |  |
| ENSG00000226380 |  | AC016831.1 |  |  | 1.82 | 6.58E-27 | DE only in female HUVECs |  |
| ENSG00000109063 |  | MYH3 |  |  | 1.78 | 2.49E-05 | DE only in female HUVECs |  |
| ENSG00000256463 |  | SALL3 |  |  | 1.63 | 3.30E-03 | DE only in female HUVECs |  |
| ENSG00000260272 |  | AC093525.2 |  |  | 1.57 | 4.20E-05 | DE only in female HUVECs | Predicted miR29a-3p target gene only |
| ENSG00000283199 |  | FP565324.1 |  |  | 1.38 | 5.65E-06 | DE only in female HUVECs |  |
| ENSG00000183785 |  | TUBA8 |  |  | 1.32 | 4.14E-03 | DE only in female HUVECs |  |
| ENSG00000260899 |  | AC106886.2 |  |  | 1.25 | 6.04E-03 | DE only in female HUVECs |  |
| ENSG00000175820 |  | CCDC168 |  |  | 1.21 | 6.78E-03 | DE only in female HUVECs |  |
| ENSG00000147174 | X | GCNA |  |  | 1.19 | 3.43E-03 | DE only in female HUVECs |  |
| ENSG00000255150 |  | EID3 |  |  | 1.03 | 3.63E-02 | DE only in female HUVECs |  |
| ENSG00000164849 |  | GPR146 |  |  | -1.11 | 4.83E-04 | DE only in female HUVECs |  |
| ENSG00000240583 |  | AQP1 |  |  | -1.16 | 8.55E-07 | DE only in female HUVECs | Predicted miR29a/c-3p common target gene |
| ENSG00000229212 |  | AC044860.1 |  |  | -1.53 | 1.79E-03 | DE only in female HUVECs |  |
| ENSG00000206652 |  | RNU1-1 |  |  | -1.71 | 1.90E-16 | DE only in female HUVECs |  |
| ENSG00000281910 |  | SNORA50A |  |  | -3.22 | 2.12E-02 | DE only in female HUVECs |  |
| ENSG00000269891 |  | ARHGAP19-SLIT1 |  |  | -4.48 | 1.94E-03 | DE only in female HUVECs |  |
| ENSG00000242735 |  | RPSAP26 |  |  | -4.95 | 1.13E-03 | DE only in female HUVECs |  |
| ENSG00000254692 |  | AL136295.1 |  |  | -5.89 | 9.84E-03 | DE only in female HUVECs |  |
| ENSG00000256514 |  | AP003419.2 |  |  | -6.1 | 4.75E-02 | DE only in female HUVECs |  |
| ENSG00000284554 |  | AL022318.4 |  |  | -6.63 | 2.98E-04 | DE only in female HUVECs |  |
| ENSG00000269749 |  | AC005614.2 |  |  | -6.78 | 5.75E-04 | DE only in female HUVECs |  |
| MiR-29c-3p(i): MiR-29c-3p inhibitor; NC: Negative Control; FDR adjusted *P*-value: Benjamini Hochberg adjusted *P*-value; DE: Differentially expressed; Bold: Differently expressed in both male and female HUVECs. *P* < 0.05 is considered significantly differentially expressed. n = 3 and 4 male and female HUVECs preparations. | | | | | | | | |
