# Supplementary material for: Sexually Dimorphic Regulation of MiR‐29a/c‐3p in Human Endothelial Cells: Cell Functions and Transcriptome
Source: J Cell Physiol. 2026 Jun 14;241(6):e70199. doi: 10.1002/jcp.70199 (PMC13266284; doi:10.1002/jcp.70199)
Supplement: Supplementary file 5 — Supporting File 5 [file JCP-241-0-s008.docx]

| Table S4. MiR-29c-3p(i)-induced diseases and biological functions in HUVECs. | | |
| --- | --- | --- |
|  | *P*-Value | *P*-Value |
|  | [Male HUVECs] | [Female HUVECs] |
| Abdominal neoplasm | **6.61E-03** | **1.54E-07** |
| Digestive organ tumor | **1.18E-02** | **1.64E-07** |
| Epithelial neoplasm | **1.18E-02** | **6.03E-07** |
| Gastrointestinal tumor | **1.18E-02** | **1.06E-05** |
| Abdominal carcinoma | **1.19E-02** | **1.64E-07** |
| Non-melanoma solid tumor | **1.28E-02** | **9.38E-07** |
| Intraabdominal organ tumor | **1.63E-02** | **1.64E-07** |
| Extracranial solid tumor | **1.63E-02** | **6.03E-07** |
| Digestive system cancer | **1.71E-02** | **8.23E-07** |
| Adenocarcinoma | **1.71E-02** | **5.11E-06** |
| Gastrointestinal adenocarcinoma | **1.71E-02** | **6.33E-05** |
| Large intestine neoplasm | **1.71E-02** | **8.51E-04** |
| Colorectal tumor | **1.71E-02** | **1.56E-02** |
| Non-colon gastrointestinal cancer | **1.71E-02** | **2.49E-02** |
| Adhesion of myeloid cells in melanoma | **1.71E-02** | **3.71E-02** |
| Morphology of basophilic leukemia cells | **1.71E-02** | **3.71E-02** |
| Oxidation of histamine | **1.71E-02** | **3.71E-02** |
| Regression of colorectal carcinoma | **1.71E-02** | **3.71E-02** |
| Premature senescence of stomach cancer cell lines | **1.71E-02** | **3.71E-02** |
| Severe essential hypertension | **1.71E-02** | **3.71E-02** |
| Structure of elastic lamina | **1.71E-02** | **3.71E-02** |
| Regulation of histamine | **1.71E-02** | **3.71E-02** |
| Upper abdominal cancer | **1.92E-02** | **1.80E-05** |
| Extrapancreatic malignant tumor | **2.12E-02** | **2.59E-06** |
| Abdominal adenocarcinoma | **2.18E-02** | **5.30E-06** |
| Cell rolling of Th2 cells | **2.37E-02** | **4.93E-04** |
| Malignant genitourinary solid tumor | **2.37E-02** | **6.21E-04** |
| Colorectal cancer | **2.50E-02** | **2.16E-02** |
| Upper gastrointestinal carcinoma | **2.86E-02** | **1.72E-02** |
| Large intestine carcinoma | **3.49E-02** | **3.98E-04** |
| Colon tumor | **3.49E-02** | **1.38E-03** |
| Genitourinary carcinoma | **3.66E-02** | **6.21E-04** |
| Head and neck squamous cell carcinoma | **4.03E-02** | **2.53E-03** |
| Laser induced choroidal neovascularization | **4.49E-02** | **1.90E-02** |
| Development of digestive organ tumor | **4.62E-02** | **5.10E-04** |
| Breast or gastric cancer | **4.75E-02** | **6.28E-03** |
| Breast or colorectal cancer | **4.75E-02** | **2.32E-02** |
| Large intestine adenocarcinoma | **4.92E-02** | **6.21E-04** |
| Vascularization of epithelial tissue | **4.20E-03** | 1.00E+00 |
| Capillarization | **4.20E-03** | 1.00E+00 |
| Quantity of myeloid cells | **4.20E-03** | 1.00E+00 |
| Function of T lymphocytes | **6.61E-03** | 1.00E+00 |
| Quantity of innate lymphoid cells | **6.61E-03** | 1.00E+00 |
| Trafficking of leukocytes | **8.61E-03** | 1.00E+00 |
| Polarization of Th1 cells | **1.18E-02** | 1.30E-01 |
| Morphology of tumor | **1.18E-02** | 1.00E+00 |
| Vascularization | **1.18E-02** | 1.00E+00 |
| Eosinophilia | **1.28E-02** | 1.00E+00 |
| Quantity of natural killer T lymphocytes | **1.28E-02** | 1.00E+00 |
| Degranulation of leukocytes | **1.28E-02** | 1.00E+00 |
| Frequency of Th17 cells | **1.28E-02** | 1.00E+00 |
| Morphology of lesion | **1.28E-02** | 1.00E+00 |
| Conduction of heart | **1.63E-02** | 1.00E+00 |
| Quantity of leukocytes | **1.63E-02** | 1.00E+00 |
| Eosinophilic inflammation | **1.71E-02** | 1.26E-01 |
| tumor microenvironment of metastasis | **1.71E-02** | 1.00E+00 |
| Recruitment of lymphocytes | **1.71E-02** | 1.00E+00 |
| Trafficking of T lymphocytes | **1.71E-02** | 1.00E+00 |
| Cell death of immune cells | **1.71E-02** | 1.00E+00 |
| Leukopoiesis | **1.71E-02** | 1.00E+00 |
| Development of antigen presenting cells | **1.71E-02** | 1.00E+00 |
| Gastrointestinal tract cancer | **1.71E-02** | 1.00E+00 |
| Arrest in differentiation of hematopoietic cell lines | **1.71E-02** | 1.00E+00 |
| Morphogenesis of acinus | **1.71E-02** | 1.00E+00 |
| Arrest in differentiation of granulocytes | **1.71E-02** | 1.00E+00 |
| Depletion of water | **1.71E-02** | 1.00E+00 |
| Paracellular permeability of gastric epithelial cells | **1.71E-02** | 1.00E+00 |
| Arrest in differentiation of bone marrow cell lines | **1.71E-02** | 1.00E+00 |
| Endomitosis of monocytes | **1.71E-02** | 1.00E+00 |
| Chemokinesis of monocytes | **1.71E-02** | 1.00E+00 |
| Autosomal dominant mental retardation type 38 | **1.71E-02** | 1.00E+00 |
| Survival of organism | **1.71E-02** | 1.00E+00 |
| Frequency of T lymphocytes | **1.71E-02** | 1.00E+00 |
| Delay in degeneration of CA1 neuron | **1.71E-02** | 1.00E+00 |
| Subcutaneous carcinoma | **1.71E-02** | 1.00E+00 |
| Excitation of projection neurons | **1.71E-02** | 1.00E+00 |
| Apoptosis of leukocytes | **1.71E-02** | 1.00E+00 |
| Development of phagocytes | **1.71E-02** | 1.00E+00 |
| Early-onset severe spinocerebellar ataxia-42 with neurodevelopmental deficits | **1.71E-02** | 1.00E+00 |
| Apoptosis of T lymphocytes | **1.71E-02** | 1.00E+00 |
| Mesenteric carcinoma | **1.71E-02** | 1.00E+00 |
| Delay in initiation of disappearance of thymocytes | **1.71E-02** | 1.00E+00 |
| Cellularity of natural killer T lymphocytes | **1.71E-02** | 1.00E+00 |
| Accumulation of T lymphocytes | **1.71E-02** | 1.00E+00 |
| Quantity of natural killer cells | **1.71E-02** | 1.00E+00 |
| Accumulation of double-positive thymocyte | **1.71E-02** | 1.00E+00 |
| Deacetylation of chromatin | **1.71E-02** | 1.00E+00 |
| Apoptosis of single positive thymocytes | **1.71E-02** | 1.00E+00 |
| Early infantile epileptic encephalopathy type 33 | **1.71E-02** | 1.00E+00 |
| Portal hypertension | **1.81E-02** | 1.00E+00 |
| Abnormal morphology of myeloid progenitor cells | **1.99E-02** | 1.00E+00 |
| Upper gastrointestinal tract tumor | **2.06E-02** | 1.00E+00 |
| Quantity of phagocytes | **2.12E-02** | 1.00E+00 |
| Respiratory burst of phagocytes | **2.18E-02** | 1.00E+00 |
| Failure of heart | **2.22E-02** | 1.14E-01 |
| Cell flattening of neuroblastoma cell lines | **2.37E-02** | 5.97E-02 |
| Premature senescence of prostate cancer cell lines | **2.37E-02** | 5.97E-02 |
| Survival of BMMC cells | **2.37E-02** | 5.97E-02 |
| Emigration of leukocytes | **2.37E-02** | 1.05E-01 |
| Noncirrhotic portal hypertension | **2.37E-02** | 1.00E+00 |
| Trafficking of natural killer T lymphocytes | **2.37E-02** | 1.00E+00 |
| Mass of colon | **2.37E-02** | 1.00E+00 |
| Depletion of goblet cells | **2.37E-02** | 1.00E+00 |
| NISCH syndrome | **2.37E-02** | 1.00E+00 |
| Depletion of gastrointestional brush cells | **2.37E-02** | 1.00E+00 |
| Quantity of cells | **2.37E-02** | 1.00E+00 |
| Autosomal recessive spastic paraplegia type 46 | **2.37E-02** | 1.00E+00 |
| Development of hematopoietic system | **2.37E-02** | 1.00E+00 |
| Respiratory burst of myeloid cells | **2.37E-02** | 1.00E+00 |
| Migration of cerebellar granule neuron precursor cells | **2.37E-02** | 1.00E+00 |
| Delay in initiation of atrophy of gastrocnemius | **2.37E-02** | 1.00E+00 |
| Migration of peritoneal neutrophils | **2.37E-02** | 1.00E+00 |
| Nuclear type 36 mitochondrial complex I deficiency | **2.37E-02** | 1.00E+00 |
| Nodal diffuse large B-cell lymphoma | **2.37E-02** | 1.00E+00 |
| Degranulation of myeloid cells | **2.37E-02** | 1.00E+00 |
| Degranulation of phagocytes | **2.37E-02** | 1.00E+00 |
| Proliferation of epithelial cells | **2.45E-02** | 1.00E+00 |
| Quantity of reticulocytes | **2.50E-02** | 1.00E+00 |
| Quantity of antigen presenting cells | **2.76E-02** | 1.00E+00 |
| Quantity of mononuclear leukocytes | **2.79E-02** | 1.00E+00 |
| Adhesion of Th2 cells | **3.04E-02** | 7.66E-02 |
| Aggressive mature B-cell lymphoma | **3.04E-02** | 1.30E-01 |
| Angiogenesis | **3.04E-02** | 1.00E+00 |
| Clustering of follicular dendritic cells | **3.04E-02** | 1.00E+00 |
| Activation of CD19+ B lymphocytes | **3.04E-02** | 1.00E+00 |
| Activation of CD3 positive T-lymphocytes | **3.04E-02** | 1.00E+00 |
| Erosion of calvaria | **3.04E-02** | 1.00E+00 |
| Inflammation of gastrointestinal tract | **3.04E-02** | 1.00E+00 |
| Trafficking of hematopoietic stem cells | **3.04E-02** | 1.00E+00 |
| Recruitment of natural killer T lymphocytes | **3.04E-02** | 1.00E+00 |
| Destruction of alveolar bone | **3.04E-02** | 1.00E+00 |
| Malignant neoplasm of large intestine | **3.04E-02** | 1.00E+00 |
| Cell-mediated response | **3.05E-02** | 1.00E+00 |
| Cell viability of lymphatic system cells | **3.08E-02** | 1.00E+00 |
| Eosinophilia of esophagus | **3.14E-02** | 1.30E-01 |
| Hereditary polyneuropathy | **3.21E-02** | 1.00E+00 |
| Mass of adipose tissue | **3.28E-02** | 1.00E+00 |
| Inflammation of respiratory system component | **3.37E-02** | 1.00E+00 |
| Function of helper T lymphocytes | **3.49E-02** | 1.00E+00 |
| Cerebrovascular dysfunction | **3.49E-02** | 1.00E+00 |
| Apoptosis of phagocytes | **3.49E-02** | 1.00E+00 |
| Transendothelial migration of regulatory T lymphocytes | **3.49E-02** | 9.36E-02 |
| Adhesion of high endothelial postcapillary venule | **3.49E-02** | 1.05E-01 |
| Abnormal morphology of Th2 cells | **3.49E-02** | 1.05E-01 |
| Disorganization of lymphoid tissue | **3.49E-02** | 1.05E-01 |
| Function of lacrimal gland | **3.49E-02** | 1.05E-01 |
| Production of porphyrin | **3.49E-02** | 1.05E-01 |
| Primary neuroblastoma | **3.49E-02** | 1.05E-01 |
| Stomach tumor | **3.49E-02** | 1.00E+00 |
| Atrial hypertrophy | **3.49E-02** | 1.00E+00 |
| Nephrotoxic acute renal failure | **3.49E-02** | 1.00E+00 |
| Interaction of leukocytes | **3.49E-02** | 1.00E+00 |
| Size of atrium | **3.49E-02** | 1.00E+00 |
| Cell rolling of leukocytes | **3.49E-02** | 1.00E+00 |
| Vasculogenesis | **3.49E-02** | 1.00E+00 |
| Tubulogenesis of endothelial cell lines | **3.49E-02** | 1.00E+00 |
| Permeabilization of tight junctions | **3.49E-02** | 1.00E+00 |
| Morphology of lymphatic system cells | **3.49E-02** | 1.00E+00 |
| Endocytosis by monocytes | **3.49E-02** | 1.00E+00 |
| Degranulation of CD8+ T lymphocyte | **3.49E-02** | 1.00E+00 |
| Effector phase of CD8+ T lymphocyte | **3.49E-02** | 1.00E+00 |
| Polarization of mitochondrial membrane | **3.49E-02** | 1.00E+00 |
| Proliferation of CD19+ B lymphocytes | **3.49E-02** | 1.00E+00 |
| Recurrent activated B-cell-like diffuse large B-cell lymphoma | **3.49E-02** | 1.00E+00 |
| Redistribution of membrane rafts | **3.49E-02** | 1.00E+00 |
| Refractory germinal center B-cell-like diffuse large B-cell lymphoma | **3.49E-02** | 1.00E+00 |
| Development of plasmacytoid precursor dendritic cells | **3.49E-02** | 1.00E+00 |
| Relapsed germinal center B-cell-like diffuse large B-cell lymphoma | **3.49E-02** | 1.00E+00 |
| Morphology of blood cells | **3.49E-02** | 1.00E+00 |
| Morphology of myeloid cells | **3.49E-02** | 1.00E+00 |
| Recruitment of T lymphocytes | **3.49E-02** | 1.00E+00 |
| Apoptosis of myeloid cells | **3.49E-02** | 1.00E+00 |
| Activation of neutrophils in lung | **3.49E-02** | 1.00E+00 |
| Abnormal quantity of leukocytes | **3.49E-02** | 1.00E+00 |
| Chemotaxis of peritoneal neutrophils | **3.49E-02** | 1.00E+00 |
| Apoptosis of muscle cell lines | **3.49E-02** | 1.00E+00 |
| Entry into cell cycle progression of gdT17 cells | **3.49E-02** | 1.00E+00 |
| Size of posterior semicircular canal | **3.49E-02** | 1.00E+00 |
| Formation of vestibule | **3.49E-02** | 1.00E+00 |
| Size of superior semicircular canal | **3.49E-02** | 1.00E+00 |
| Antiviral response of kidney cell lines | **3.49E-02** | 1.00E+00 |
| Lack of horizontal semicircular canal | **3.49E-02** | 1.00E+00 |
| Frequency of gamma-delta T lymphocytes | **3.49E-02** | 1.00E+00 |
| Inflammation of peritoneum | **3.49E-02** | 1.00E+00 |
| Lack of vestibule | **3.49E-02** | 1.00E+00 |
| Quantity of gdT17 cells | **3.49E-02** | 1.00E+00 |
| Quantity of TREG cells | **3.60E-02** | 1.00E+00 |
| Growth of melanoma | **3.65E-02** | 1.00E+00 |
| Premature senescence of breast cancer cell lines | **3.66E-02** | 1.16E-01 |
| Regression of tumor | **3.66E-02** | 1.00E+00 |
| Rejection of malignant tumor | **3.66E-02** | 1.00E+00 |
| tumor burden | **3.66E-02** | 1.00E+00 |
| Establishment of blood-brain barrier | **3.66E-02** | 1.00E+00 |
| Trafficking of B lymphocytes | **3.66E-02** | 1.00E+00 |
| Reassembly of tight junctions | **3.66E-02** | 1.00E+00 |
| Trafficking of neutrophils | **3.66E-02** | 1.00E+00 |
| Endotoxin shock response | **3.66E-02** | 1.00E+00 |
| Epilepsy | **3.66E-02** | 1.00E+00 |
| Loss of natural killer cells | **3.66E-02** | 1.00E+00 |
| Cell survival of leukemia cell lines | **3.66E-02** | 1.00E+00 |
| Regeneration of peripheral nerve | **3.66E-02** | 1.00E+00 |
| Quantity of IgG2a | **3.66E-02** | 1.00E+00 |
| Lack of posterior semicircular canal | **3.66E-02** | 1.00E+00 |
| Antiviral response of epithelial cell lines | **3.66E-02** | 1.00E+00 |
| Analgesia | **3.66E-02** | 1.00E+00 |
| Familial amyloid polyneuropathy type I | **3.66E-02** | 1.00E+00 |
| Activation of M1 macrophages | **3.66E-02** | 1.00E+00 |
| Degeneration of macula of saccule | **3.66E-02** | 1.00E+00 |
| Cell death of hippocampal neurons | **3.66E-02** | 1.00E+00 |
| Delay in apoptosis of embryonic cell lines | **3.66E-02** | 1.00E+00 |
| Cell viability of leukocytes | **3.78E-02** | 1.00E+00 |
| Quantity of Ca2+ | **3.93E-02** | 1.00E+00 |
| Flow of urine | **3.95E-02** | 1.28E-01 |
| Chemotaxis of germ cell tumor cell lines | **3.95E-02** | 1.00E+00 |
| Histopathological change of liver | **3.95E-02** | 1.00E+00 |
| Quantity of immunoglobulin | **3.95E-02** | 1.00E+00 |
| Primary thyroid gland neoplasm | **3.95E-02** | 1.00E+00 |
| Delay in apoptosis of fibroblast cell lines | **3.95E-02** | 1.00E+00 |
| Delay in amyotrophic lateral sclerosis | **3.95E-02** | 1.00E+00 |
| Degeneration of macula of utricle | **3.95E-02** | 1.00E+00 |
| Newly diagnosed chronic lymphocytic leukemia | **3.95E-02** | 1.00E+00 |
| Pulmonary nodule | **3.95E-02** | 1.00E+00 |
| Infection by Bacilli | **3.95E-02** | 1.00E+00 |
| Maturation of bone marrow-derived mast cells | **3.95E-02** | 1.00E+00 |
| Apoptosis of superior cervical ganglion neurons | **3.95E-02** | 1.00E+00 |
| Mobilization of myeloid progenitor cells | **3.95E-02** | 1.00E+00 |
| Inflammation of organ | **3.98E-02** | 1.00E+00 |
| Organismal death | **4.13E-02** | 1.00E+00 |
| Abnormal morphology of Th1 cells | **4.17E-02** | 1.30E-01 |
| Visceral leishmaniasis | **4.17E-02** | 1.30E-01 |
| Acquired generalized hypoactive sexual desire disorder | **4.17E-02** | 1.30E-01 |
| Size of tumor | **4.17E-02** | 1.00E+00 |
| Conduction of atrioventricular node | **4.17E-02** | 1.00E+00 |
| Conduction of sinoatrial node | **4.17E-02** | 1.00E+00 |
| Abnormal morphology of leukocytes | **4.17E-02** | 1.00E+00 |
| Expression of autoantibody | **4.17E-02** | 1.00E+00 |
| Quantity of adipoblasts | **4.17E-02** | 1.00E+00 |
| Proliferation of CD3 positive T-lymphocytes | **4.17E-02** | 1.00E+00 |
| Gastroesophageal cancer | **4.17E-02** | 1.00E+00 |
| Chemokinesis of neutrophils | **4.17E-02** | 1.00E+00 |
| Malignant neoplastic nodule | **4.17E-02** | 1.00E+00 |
| Development of macrophages | **4.17E-02** | 1.00E+00 |
| Activation of neutrophils | **4.17E-02** | 1.00E+00 |
| Size of lesion | **4.19E-02** | 1.00E+00 |
| Diffuse large B-cell lymphoma | **4.19E-02** | 1.00E+00 |
| Weight loss | **4.19E-02** | 1.00E+00 |
| Autosomal dominant polycystic kidney disease | **4.32E-02** | 1.00E+00 |
| Necrosis of liver | **4.36E-02** | 1.00E+00 |
| Onset of insulin-dependent diabetes mellitus | **4.49E-02** | 1.00E+00 |
| Heart rate | **4.49E-02** | 1.00E+00 |
| Polarization of PBMCs | **4.49E-02** | 1.00E+00 |
| Abnormal morphology of megakaryocyte/erythrocyte lineage-restricted progenitor cells | **4.49E-02** | 1.00E+00 |
| Development of hematopoietic cells | **4.49E-02** | 1.00E+00 |
| Abnormal morphology of ampullary crest | **4.49E-02** | 1.00E+00 |
| Cell viability of phagocytes | **4.53E-02** | 1.00E+00 |
| Activation of leukocytes | **4.57E-02** | 1.00E+00 |
| Primary neoplasm | **4.58E-02** | 1.00E+00 |
| Abnormal morphology of T lymphocytes | **4.62E-02** | 1.00E+00 |
| Cell viability of myeloid cells | **4.62E-02** | 1.00E+00 |
| Visceromegaly of organ | **4.68E-02** | 1.30E-01 |
| Penetrance of tumor | **4.68E-02** | 1.00E+00 |
| Dysfunction of liver | **4.68E-02** | 1.00E+00 |
| Seizures | **4.68E-02** | 1.00E+00 |
| Function of memory T lymphocytes | **4.68E-02** | 1.00E+00 |
| Respiratory burst of monocytes | **4.68E-02** | 1.00E+00 |
| Relapsed chronic lymphocytic leukemia | **4.68E-02** | 1.00E+00 |
| Experimentally induced bacterial peritonitis | **4.68E-02** | 1.00E+00 |
| Cell viability of T lymphocytes | **4.68E-02** | 1.00E+00 |
| Quantity of CD11b+ cells | **4.75E-02** | 1.30E-01 |
| Growth of malignant tumor | **4.75E-02** | 1.00E+00 |
| Pressure overload hypertrophy | **4.75E-02** | 1.00E+00 |
| Quantity of lymphocytes | **4.75E-02** | 1.00E+00 |
| Abnormal morphology of spleen | **4.75E-02** | 1.00E+00 |
| Tolerization | **4.75E-02** | 1.00E+00 |
| Discriminatory learning | **4.75E-02** | 1.00E+00 |
| Apoptosis of invariant natural killer T cells | **4.75E-02** | 1.00E+00 |
| Cell viability of monocytes | **4.75E-02** | 1.00E+00 |
| Morphology of cytotoxic T cells | **4.75E-02** | 1.00E+00 |
| Migration of airway smooth muscle cells | **4.75E-02** | 1.00E+00 |
| Quantity of phosphatidylinositol 4-phosphate | **4.75E-02** | 1.00E+00 |
| Proliferation of glioma stem cells | **4.75E-02** | 1.00E+00 |
| Survival of Staphylococcus aureus | **4.75E-02** | 1.00E+00 |
| Differentiation of natural T-regulatory cells | **4.75E-02** | 1.00E+00 |
| Transformation of melanocytes | **4.75E-02** | 1.00E+00 |
| Apoptosis of bone marrow-derived mast cells | **4.75E-02** | 1.00E+00 |
| G1/S phase transition of fibroblasts | **4.75E-02** | 1.00E+00 |
| Elevation of Ca2+ in cytosol | **4.75E-02** | 1.00E+00 |
| Accumulation of Th17 cells | **4.75E-02** | 1.00E+00 |
| Damage of digestive system | **4.79E-02** | 1.00E+00 |
| Gastric cancer | **4.80E-02** | 1.00E+00 |
| Activation of cells | **4.85E-02** | 1.00E+00 |
| Peripheral neuropathy | **4.85E-02** | 9.99E-02 |
| Visceromegaly | **4.91E-02** | 1.00E+00 |
| Development of connective tissue cells | **4.91E-02** | 1.00E+00 |
| Relapsed lymphocytic cancer | **4.91E-02** | 1.00E+00 |
| Paired-pulse inhibition | **4.92E-02** | 1.30E-01 |
| Colon cancer | **4.92E-02** | 1.00E+00 |
| Immune response of lymph node cells | **4.92E-02** | 1.00E+00 |
| Proliferation of lymphatic system cells | **4.92E-02** | 1.00E+00 |
| Apoptosis of peripheral T lymphocyte | **4.92E-02** | 1.00E+00 |
| Leukocytosis | **4.92E-02** | 1.00E+00 |
| Colony survival of lung cancer cell lines | **4.92E-02** | 1.00E+00 |
| Recurrent lymphocytic cancer | **4.92E-02** | 1.00E+00 |
| Adhesion of bone marrow-derived macrophages | **4.92E-02** | 1.00E+00 |
| Cell death of pheochromocytoma cell lines | **4.92E-02** | 1.00E+00 |
| T cell development | **4.92E-02** | 1.00E+00 |
| Proliferation of retinoblastoma cell lines | **4.92E-02** | 1.00E+00 |
| Function of immune system | 5.10E-02 | **3.33E-02** |
| Head and neck carcinoma | 5.15E-02 | **1.09E-06** |
| Gastroesophageal adenocarcinoma | 5.24E-02 | **4.43E-03** |
| Gastro-esophageal carcinoma | 5.30E-02 | **3.66E-03** |
| Squamous-cell carcinoma | 5.55E-02 | **4.43E-03** |
| Thyroid carcinoma | 5.74E-02 | **2.81E-06** |
| Extrapulmonary squamous cell carcinoma | 5.87E-02 | **5.74E-03** |
| Skin lesion | 6.06E-02 | **1.50E-04** |
| tumorigenesis of epithelial neoplasm | 6.18E-02 | **1.43E-04** |
| Gastric carcinoma | 6.31E-02 | **3.87E-03** |
| Cancer of cells | 6.35E-02 | **3.33E-04** |
| Gastric adenocarcinoma | 6.48E-02 | **3.72E-02** |
| Head and neck tumor | 6.58E-02 | **7.62E-07** |
| Frequency of tumor | 6.58E-02 | **2.04E-04** |
| Mature lymphocytic neoplasm | 6.88E-02 | **7.05E-03** |
| Upper aerodigestive tract carcinoma | 7.02E-02 | **3.37E-03** |
| Cancer of secretory structure | 7.26E-02 | **2.59E-06** |
| Hematologic cancer of cells | 7.66E-02 | **5.57E-03** |
| Incidence of tumor | 7.71E-02 | **2.09E-04** |
| Malignant neoplasm of respiratory system | 8.35E-02 | **1.50E-04** |
| Multiorgan inflammation | 8.35E-02 | **5.13E-03** |
| Development of carcinoma | 8.88E-02 | **2.15E-04** |
| Skin cancer | 8.88E-02 | **6.18E-04** |
| Lung lesion | 8.88E-02 | **2.04E-03** |
| Esophageal lesion | 8.93E-02 | **1.16E-02** |
| Cutaneous melanoma | 8.95E-02 | **1.50E-04** |
| Neoplasia of blood cells | 8.99E-02 | **3.71E-02** |
| Neoplasia of leukocytes | 9.09E-02 | **1.28E-02** |
| Hepato-pancreato-biliary cancer | 9.22E-02 | **4.07E-06** |
| Abdominal cancer | 1.00E+00 | **1.64E-07** |
| Head and neck cancer | 1.00E+00 | **6.03E-07** |
| Nonpituitary endocrine tumor | 1.00E+00 | **9.38E-07** |
| Thyroid gland tumor | 1.00E+00 | **1.18E-06** |
| Endocrine gland tumor | 1.00E+00 | **2.14E-06** |
| Carcinoma | 1.00E+00 | **2.28E-06** |
| Non-central nervous system malignant neoplasm | 1.00E+00 | **2.59E-06** |
| Endocrine carcinoma | 1.00E+00 | **2.88E-06** |
| Malignant solid tumor | 1.00E+00 | **3.64E-06** |
| Gastrointestinal carcinoma | 1.00E+00 | **3.87E-06** |
| Melanoma | 1.00E+00 | **7.56E-06** |
| Hepatobiliary neoplasm | 1.00E+00 | **8.61E-05** |
| Formation of solid tumor | 1.00E+00 | **1.24E-04** |
| T-cell malignant neoplasm | 1.00E+00 | **1.24E-04** |
| Neurogenic lesion | 1.00E+00 | **1.69E-04** |
| Gliomatosis cerebri | 1.00E+00 | **1.73E-04** |
| Genitourinary adenocarcinoma | 1.00E+00 | **2.04E-04** |
| Brain lesion | 1.00E+00 | **3.01E-04** |
| Brain glioma | 1.00E+00 | **3.29E-04** |
| Generation of tumor | 1.00E+00 | **3.62E-04** |
| Grade 3-4 tumor | 1.00E+00 | **4.01E-04** |
| Hepatobiliary carcinoma | 1.00E+00 | **4.50E-04** |
| Intrathoracic malignant tumor | 1.00E+00 | **4.59E-04** |
| Brain astrocytoma | 1.00E+00 | **4.93E-04** |
| Grade 4 high grade glioma | 1.00E+00 | **4.93E-04** |
| Grade 4 astrocytoma | 1.00E+00 | **4.93E-04** |
| T-cell prolymphocytic leukemia | 1.00E+00 | **4.93E-04** |
| Liver tumor | 1.00E+00 | **5.84E-04** |
| Lymphocytic cancer | 1.00E+00 | **6.21E-04** |
| Malignant lymphocytic neoplasm | 1.00E+00 | **6.21E-04** |
| Mature T-cell neoplasm | 1.00E+00 | **6.29E-04** |
| Grade 3-4 glioma cancer | 1.00E+00 | **6.50E-04** |
| Lung carcinoma | 1.00E+00 | **6.50E-04** |
| Pelvic cancer | 1.00E+00 | **6.50E-04** |
| Lung cancer | 1.00E+00 | **8.40E-04** |
| Colon carcinoma | 1.00E+00 | **8.51E-04** |
| Central nervous system cancer | 1.00E+00 | **9.53E-04** |
| Genital tract cancer | 1.00E+00 | **1.02E-03** |
| Central nervous system solid tumor | 1.00E+00 | **1.10E-03** |
| Pelvic carcinoma | 1.00E+00 | **1.21E-03** |
| Glioma cancer | 1.00E+00 | **1.29E-03** |
| Genital carcinoma | 1.00E+00 | **1.45E-03** |
| Blue round small cell tumor | 1.00E+00 | **1.56E-03** |
| Genital tumor | 1.00E+00 | **1.56E-03** |
| T-cell leukemia | 1.00E+00 | **1.88E-03** |
| Pelvic adenocarcinoma | 1.00E+00 | **1.88E-03** |
| Malignant neuroendocrine neoplasm | 1.00E+00 | **2.04E-03** |
| Pancreatic carcinoma | 1.00E+00 | **2.10E-03** |
| Pancreaticobiliary carcinoma | 1.00E+00 | **2.53E-03** |
| Colon adenocarcinoma | 1.00E+00 | **3.05E-03** |
| Breast or pancreatic cancer | 1.00E+00 | **3.13E-03** |
| Development of adenocarcinoma | 1.00E+00 | **3.50E-03** |
| Chronic lymphocytic leukemia or small lymphocytic lymphoma or prolymphocytic leukemia | 1.00E+00 | **3.57E-03** |
| Small-cell carcinoma | 1.00E+00 | **3.66E-03** |
| Laryngeal squamous cell carcinoma | 1.00E+00 | **3.93E-03** |
| Skin carcinoma | 1.00E+00 | **4.41E-03** |
| Pancreaticobiliary neoplasm | 1.00E+00 | **4.91E-03** |
| Neuroendocrine tumor | 1.00E+00 | **5.34E-03** |
| Upper airway cancer | 1.00E+00 | **6.28E-03** |
| Pancreatic tumor | 1.00E+00 | **6.77E-03** |
| Female genital tract cancer | 1.00E+00 | **6.77E-03** |
| Lymphocytic leukemia | 1.00E+00 | **6.86E-03** |
| Pancreatic ductal adenocarcinoma | 1.00E+00 | **7.50E-03** |
| Liver carcinoma | 1.00E+00 | **8.03E-03** |
| Breast or ovarian carcinoma | 1.00E+00 | **8.29E-03** |
| Hematologic cancer | 1.00E+00 | **1.03E-02** |
| tumorigenesis of reproductive tract | 1.00E+00 | **1.09E-02** |
| Leukemia | 1.00E+00 | **1.15E-02** |
| Myeloid or lymphoid neoplasm | 1.00E+00 | **1.16E-02** |
| Myasthenic syndrome | 1.00E+00 | **1.19E-02** |
| Development of genital tumor | 1.00E+00 | **1.21E-02** |
| Esophageal carcinoma | 1.00E+00 | **1.28E-02** |
| Grade 3 malignant glioma | 1.00E+00 | **1.47E-02** |
| Cancer of head | 1.00E+00 | **1.47E-02** |
| Malignant neoplasm of retroperitoneum | 1.00E+00 | **1.47E-02** |
| Extrapancreatic neuroendocrine tumor | 1.00E+00 | **1.56E-02** |
| Basal cell carcinoma | 1.00E+00 | **1.56E-02** |
| Prostatic adenocarcinoma | 1.00E+00 | **1.57E-02** |
| Postsynaptic congenital myasthenic syndrome | 1.00E+00 | **1.58E-02** |
| Mixed Müllerian endometrial cancer | 1.00E+00 | **1.71E-02** |
| Breast or gynecological cancer | 1.00E+00 | **1.72E-02** |
| Oligodendroglioma | 1.00E+00 | **1.72E-02** |
| Female genital tract adenocarcinoma | 1.00E+00 | **1.73E-02** |
| Breast carcinoma | 1.00E+00 | **1.97E-02** |
| Bladder carcinoma | 1.00E+00 | **2.20E-02** |
| Weakness | 1.00E+00 | **2.20E-02** |
| Female genital carcinoma | 1.00E+00 | **2.20E-02** |
| Ductal carcinoma | 1.00E+00 | **2.59E-02** |
| Extraadrenal retroperitoneal tumor | 1.00E+00 | **2.70E-02** |
| Aggressive diffuse large B-cell lymphoma | 1.00E+00 | **2.72E-02** |
| Small-cell carcinoma in gallbladder | 1.00E+00 | **2.73E-02** |
| Pancreatobiliary adenocarcinoma | 1.00E+00 | **2.93E-02** |
| Brain oligodendroglioma | 1.00E+00 | **2.94E-02** |
| Binding of helper T lymphocytes | 1.00E+00 | **2.94E-02** |
| Rhabdomyolysis | 1.00E+00 | **2.94E-02** |
| Small cell lung carcinoma | 1.00E+00 | **2.95E-02** |
| Ovarian tumor | 1.00E+00 | **3.05E-02** |
| Ductal adenocarcinoma | 1.00E+00 | **3.05E-02** |
| ALK fusion positive CD20 positive diffuse large B-cell non-Hodgkin lymphoma | 1.00E+00 | **3.48E-02** |
| Malignant neoplasm of male genital organ | 1.00E+00 | **3.50E-02** |
| Malignant mixed Mullerian tumor in endometrium | 1.00E+00 | **3.71E-02** |
| Long-QT syndrome | 1.00E+00 | **3.71E-02** |
| Dilated cardiomyopathy type 1MM | 1.00E+00 | **3.71E-02** |
| Severe experimental autoimmune myocarditis | 1.00E+00 | **3.71E-02** |
| Left ventricular noncompaction type 10 | 1.00E+00 | **3.71E-02** |
| Cardiac and cutaneous amyloidosis | 1.00E+00 | **3.71E-02** |
| Familial amyloid polyneuropathy type III | 1.00E+00 | **3.71E-02** |
| Force generation of myocardium | 1.00E+00 | **3.71E-02** |
| Susceptibility to familial hypertrophic cardiomyopathy 4 | 1.00E+00 | **3.71E-02** |
| Autosomal dominant hyperkalemic periodic paralysis | 1.00E+00 | **3.71E-02** |
| Diastolic relaxation of left ventricle | 1.00E+00 | **3.71E-02** |
| Contractility of left ventricle | 1.00E+00 | **3.71E-02** |
| Proliferation of arterial capillary | 1.00E+00 | **3.71E-02** |
| Translocation of water | 1.00E+00 | **3.71E-02** |
| Cell rolling of blood platelets | 1.00E+00 | **3.71E-02** |
| Breast or ovarian neoplasm | 1.00E+00 | **3.71E-02** |
| Breast or ovarian cancer | 1.00E+00 | **3.71E-02** |
| Response of fibroblast-like synoviocytes | 1.00E+00 | **3.71E-02** |
| Freeman-sheldon syndrome | 1.00E+00 | **3.71E-02** |
| Fetal akinesia deformation sequence 2 | 1.00E+00 | **3.71E-02** |
| Depletion of sphingomyelin | 1.00E+00 | **3.71E-02** |
| Area of zone of proliferation | 1.00E+00 | **3.71E-02** |
| Recruitment of cholesterol | 1.00E+00 | **3.71E-02** |
| Autosomal recessive deafness type 3 | 1.00E+00 | **3.71E-02** |
| Removal of phosphatidylcholine | 1.00E+00 | **3.71E-02** |
| Induction of macrophage-derived foam cells | 1.00E+00 | **3.71E-02** |
| Clearance of lipopolysaccharide | 1.00E+00 | **3.71E-02** |
| Efflux of alpha-tocopherol | 1.00E+00 | **3.71E-02** |
| Autosomal dominant congenital stationary night blindness 3 | 1.00E+00 | **3.71E-02** |
| Distal arthrogryposis type 2B3 | 1.00E+00 | **3.71E-02** |
| Uncombable hair syndrome 3 | 1.00E+00 | **3.71E-02** |
| Delay in closure of suture | 1.00E+00 | **3.71E-02** |
| Recruitment of sphingomyelin | 1.00E+00 | **3.71E-02** |
| Oxidation of 1-palmitoyl-2-linoleoyl-sn-glycero-3-phosphocholine | 1.00E+00 | **3.71E-02** |
| Autosomal dominant multiple pterygium syndrome | 1.00E+00 | **3.71E-02** |
| Hearing loss with Smith Magenis syndrome | 1.00E+00 | **3.71E-02** |
| Desensitization of rod bipolar cells | 1.00E+00 | **3.71E-02** |
| Congenital myasthenic syndrome associated with acetylcholine receptor deficiency type 11 | 1.00E+00 | **3.71E-02** |
| Desensitization of retinal rods | 1.00E+00 | **3.71E-02** |
| Area of zone of hypertrophy | 1.00E+00 | **3.71E-02** |
| Spermatogenic failure 39 | 1.00E+00 | **3.71E-02** |
| Depletion of phosphatidylcholine | 1.00E+00 | **3.71E-02** |
| Autosomal recessive primary ciliary dyskinesia type 5 | 1.00E+00 | **3.71E-02** |
| Congenital stationary night blindness type 1G | 1.00E+00 | **3.71E-02** |
| Autosomal recessive glycogen storage disease V | 1.00E+00 | **3.71E-02** |
| Contractures, pterygia and variable skeletal fusions syndrome 1B | 1.00E+00 | **3.71E-02** |
| Hereditary apolipoprotein A-I deficiency | 1.00E+00 | **3.71E-02** |
| Prostate cancer | 1.00E+00 | **3.71E-02** |
| Spindle cell neoplasm | 1.00E+00 | **3.71E-02** |
| Ovarian cancer | 1.00E+00 | **3.71E-02** |
| Familial congenital myopathy | 1.00E+00 | **3.71E-02** |
| Cell rolling of pre-B lymphocytes | 1.00E+00 | **3.71E-02** |
| Agammaglobulinemia type 6 | 1.00E+00 | **3.71E-02** |
| CD20 positive refractory follicular B-cell non-Hodgkin lymphoma | 1.00E+00 | **3.71E-02** |
| Epstein Barr virus positive diffuse large B-cell lymphoma | 1.00E+00 | **3.71E-02** |
| Hydrolysis of lactose | 1.00E+00 | **3.71E-02** |
| Endometrioid carcinoma | 1.00E+00 | **3.71E-02** |
| Sudden infant death syndrome | 1.00E+00 | **3.71E-02** |
| Potassium sensitive normokalemic periodic paralysis | 1.00E+00 | **3.71E-02** |
| Hypokalemic periodic paralysis type 2 | 1.00E+00 | **3.71E-02** |
| Gallbladder carcinoma | 1.00E+00 | **3.71E-02** |
| Tongue disease | 1.00E+00 | **3.71E-02** |
| Autosomal recessive deafness type 113 | 1.00E+00 | **3.71E-02** |
| Acetazolamide-responsive congenital myasthenic syndrome | 1.00E+00 | **3.71E-02** |
| Recessive congenital myasthenic syndrome | 1.00E+00 | **3.71E-02** |
| Myotonia fluctuans | 1.00E+00 | **3.71E-02** |
| Congenital lactase deficiency | 1.00E+00 | **3.71E-02** |
| Acetazolamide-responsive myotonia | 1.00E+00 | **3.71E-02** |
| Progression of pro-B lymphocytes | 1.00E+00 | **3.71E-02** |
| Stage II-IV transformed diffuse large B-cell lymphoma | 1.00E+00 | **3.71E-02** |
| Osmotic water permeability of dorsal root ganglion cells | 1.00E+00 | **3.71E-02** |
| Catabolism of lactose | 1.00E+00 | **3.71E-02** |
| Muscle weakness | 1.00E+00 | **3.71E-02** |
| Absorption of D-fructose | 1.00E+00 | **3.71E-02** |
| Paramyotonia congenita/hyperkalemic periodic paralysis | 1.00E+00 | **3.71E-02** |
| Autosomal dominant deafness type 4B | 1.00E+00 | **3.71E-02** |
| Endometrial cancer | 1.00E+00 | **3.71E-02** |
| Polymicrogyria with optic nerve hypoplasia | 1.00E+00 | **3.71E-02** |
| Catabolism of cellobiose | 1.00E+00 | **3.71E-02** |
| CD20 positive relapsed follicular B-cell non-Hodgkin lymphoma | 1.00E+00 | **3.71E-02** |
| Upper aero-digestive squamous cell carcinoma | 1.00E+00 | **3.87E-02** |
| CD20 positive T-cell/histiocyte rich diffuse large B-cell lymphoma | 1.00E+00 | **3.87E-02** |
| Familial autoimmune disease | 1.00E+00 | **3.92E-02** |
| Regression of malignant tumor | 1.00E+00 | **4.02E-02** |
| Abdominal neuroendocrine tumor | 1.00E+00 | **4.02E-02** |
| MYC translocation positive diffuse large B-cell lymphoma | 1.00E+00 | **4.02E-02** |
| Esophageal adenocarcinoma | 1.00E+00 | **4.02E-02** |
| Metabolic myopathy | 1.00E+00 | **4.02E-02** |
| Refractory CD20 positive follicular non Hodgkin lymphoma | 1.00E+00 | **4.02E-02** |
| BCL2 rearrangement positive c-MYC rearrangement positive diffuse large B-cell lymphoma | 1.00E+00 | **4.02E-02** |
| BCL6 rearrangement positive c-MYC rearrangement positive diffuse large B-cell lymphoma | 1.00E+00 | **4.02E-02** |
| Abnormal function of immune system | 1.00E+00 | **4.06E-02** |
| Nodal lymphoma | 1.00E+00 | **4.16E-02** |
| Migration of helper T lymphocytes | 1.00E+00 | **4.22E-02** |
| Relapsed CD20 positive follicular non Hodgkin lymphoma | 1.00E+00 | **4.22E-02** |
| Mixed neoplasia | 1.00E+00 | **4.30E-02** |
| Quantity of memory T lymphocytes | 1.00E+00 | **4.57E-02** |
| Transendothelial migration of T lymphocytes | 1.00E+00 | **4.74E-02** |
| Bold *P*-values: < 0.05*. P*-value: Benjamini Hochberg adjusted *P*-value. n = 3 and 4, male and female HUVECs preparations, respectively. | | |
