# Supplementary material for: Sexually Dimorphic Regulation of MiR‐29a/c‐3p in Human Endothelial Cells: Cell Functions and Transcriptome
Source: J Cell Physiol. 2026 Jun 14;241(6):e70199. doi: 10.1002/jcp.70199 (PMC13266284; doi:10.1002/jcp.70199)
Supplement: Supplementary file 6 — Supporting File 6 [file JCP-241-0-s003.docx]

| Table S5. MiR-29c-3p(i)-induced toxicity functions in HUVECs. | | |
| --- | --- | --- |
|  | *P*-Value | *P*-Value |
|  | [Male HUVECs] | [Female HUVECs] |
| Atrial hypertrophy | **4.95E-02** | 1.00E+00 |
| Dysfunction of liver | **4.95E-02** | 1.00E+00 |
| Failure of heart | **4.95E-02** | 9.26E-02 |
| Familial amyloid polyneuropathy type I | **4.95E-02** | 1.00E+00 |
| Histopathological change of liver | **4.95E-02** | 1.00E+00 |
| Increased localization of alkaline phosphatase | **4.95E-02** | 1.00E+00 |
| Necrosis of liver | **4.95E-02** | 1.00E+00 |
| Nephrotoxic acute renal failure | **4.95E-02** | 1.00E+00 |
| Pressure overload hypertrophy | **4.95E-02** | 1.00E+00 |
| Liver carcinoma | 2.31E-01 | **1.21E-02** |
| Cardiac and cutaneous amyloidosis | 1.00E+00 | **3.49E-02** |
| Dilated cardiomyopathy type 1MM | 1.00E+00 | **3.49E-02** |
| Left ventricular noncompaction type 10 | 1.00E+00 | **3.49E-02** |
| Long-QT syndrome | 1.00E+00 | **3.49E-02** |
| Severe experimental autoimmune myocarditis | 1.00E+00 | **3.49E-02** |
| Liver tumor | 1.00E+00 | **9.08E-04** |
| Bold *P*-values: < 0.05. *P*-value: Benjamini Hochberg adjusted *P*-value. n = 3 and 4, male and female HUVECs preparations, respectively. | | |
