# Supplementary material for: Sexually Dimorphic Regulation of MiR‐29a/c‐3p in Human Endothelial Cells: Cell Functions and Transcriptome
Source: J Cell Physiol. 2026 Jun 14;241(6):e70199. doi: 10.1002/jcp.70199 (PMC13266284; doi:10.1002/jcp.70199)
Supplement: Supplementary file 7 — Supporting File 7 [file JCP-241-0-s007.docx]

| Table S6. MiR-29c-3p(i)-induced gene networks in HUVECs. | | | | |
| --- | --- | --- | --- | --- |
| Gene network | Male HUVECs | | Female HUVECs | |
|  | *P*-value | Target Genes | *P*-value | Target Genes |
| TNF-regulated genes | **1.20E-03** | AOC3, ATF3, BIRC3, CLDN1, CXCL3, EBI3, RRAD, TNFRSF9 | 1.00E+00 | N/A |
| IL1B-regulated genes | **1.28E-03** | ATF3, BIRC3, CLDN1, CXCL3, EBI3, TNFRSF9 | 1.00E+00 | N/A |
| NFkB (complex)-regulated genes | **4.23E-04** | ATF3, BIRC3, CXCL3, EBI3, TNFRSF9 | 1.00E+00 | N/A |
| Bold: *P*-value<0.05; *P*-value: Benjamini Hochberg adjusted *P*-value; Z Scores > \|2\| are predicted to be target genes; N/A: prediction not available. n = 3 and 4, male and female HUVECs preparations, respectively. | | | | |
