# Supplementary material for: Sexually Dimorphic Regulation of MiR‐29a/c‐3p in Human Endothelial Cells: Cell Functions and Transcriptome
Source: J Cell Physiol. 2026 Jun 14;241(6):e70199. doi: 10.1002/jcp.70199 (PMC13266284; doi:10.1002/jcp.70199)
Supplement: Supplementary file 8 — Supporting File 8 [file JCP-241-0-s001.docx]

| Table S7. Predicted binding sites within the 3′ UTRs of miR-29-3p target genes in female HUVECs and their evolutionary conservation across species. | | |
| --- | --- | --- |
| Gene | Predicted binding sites in vertebrate species | Predicted binding sites in miR-29-3p family |
| CILP2 | 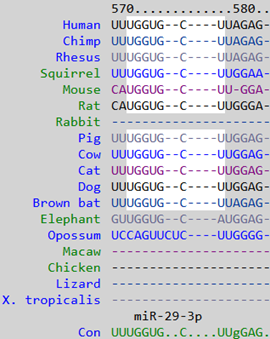 | 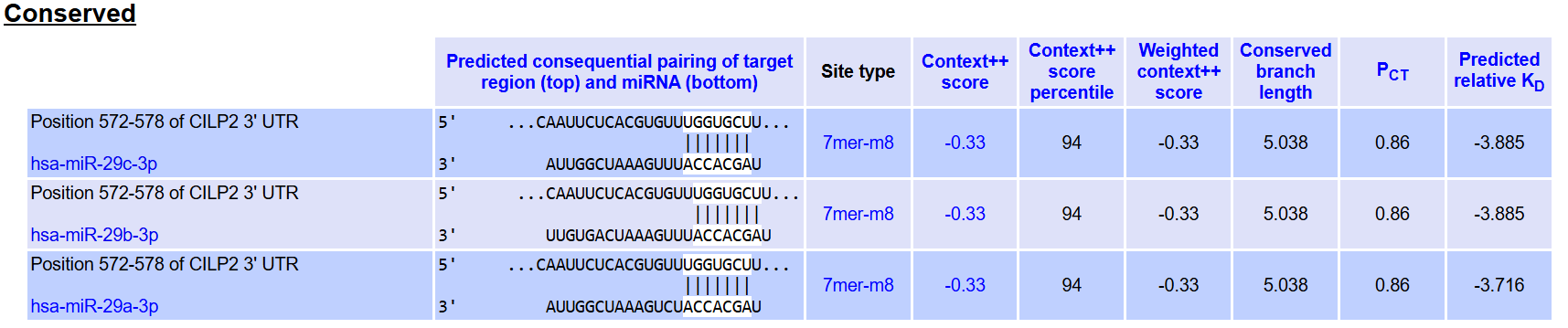 |
| ALM2-AKAP2 | 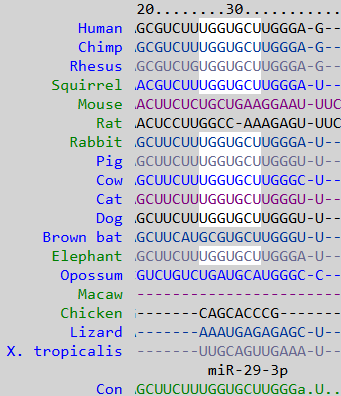 | 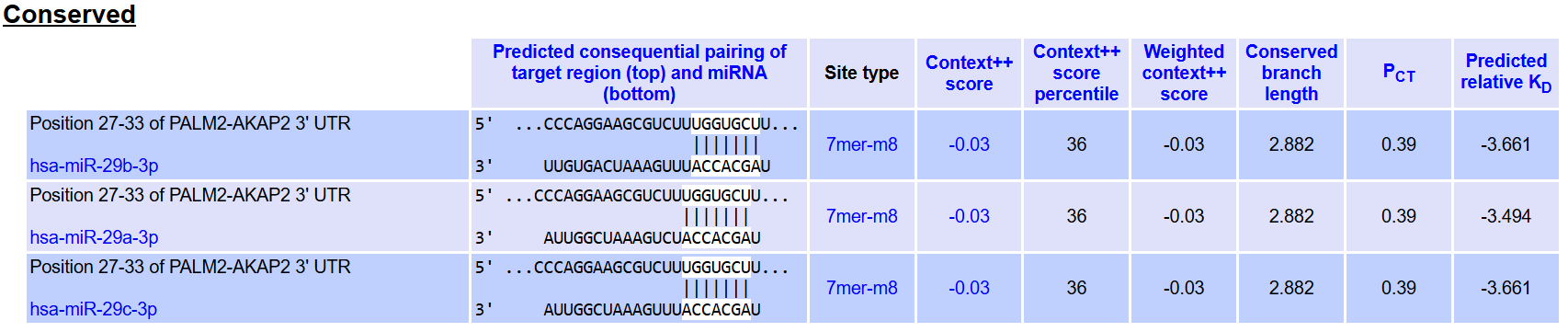 |
| TMEM256-PLSCR3 | 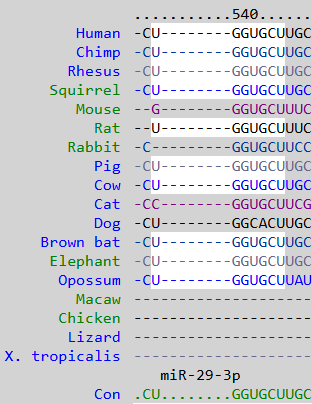 | 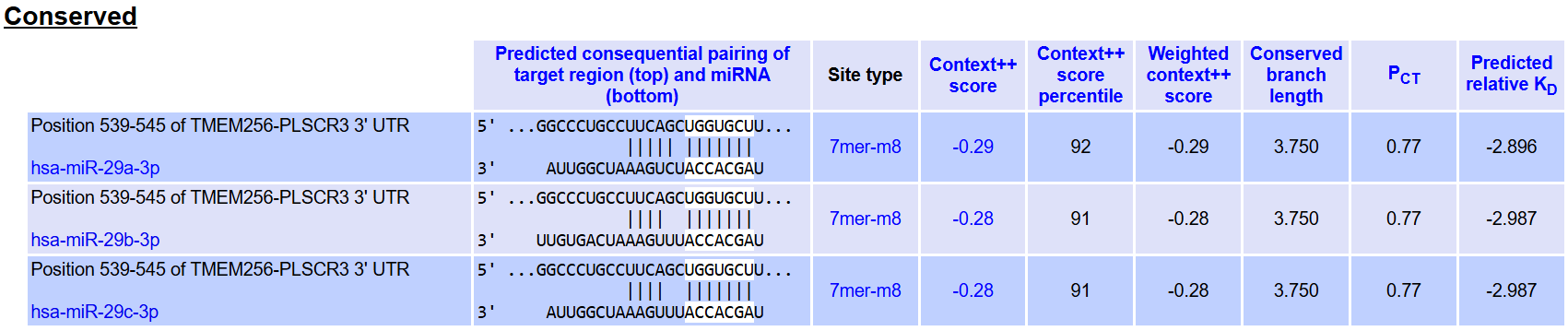 |
| CLDN1 | 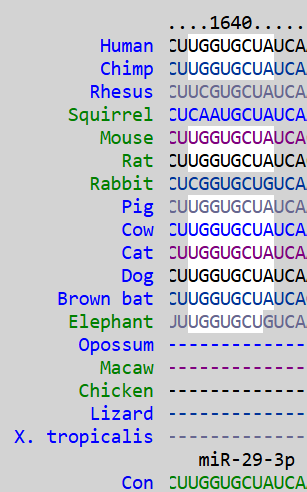 | 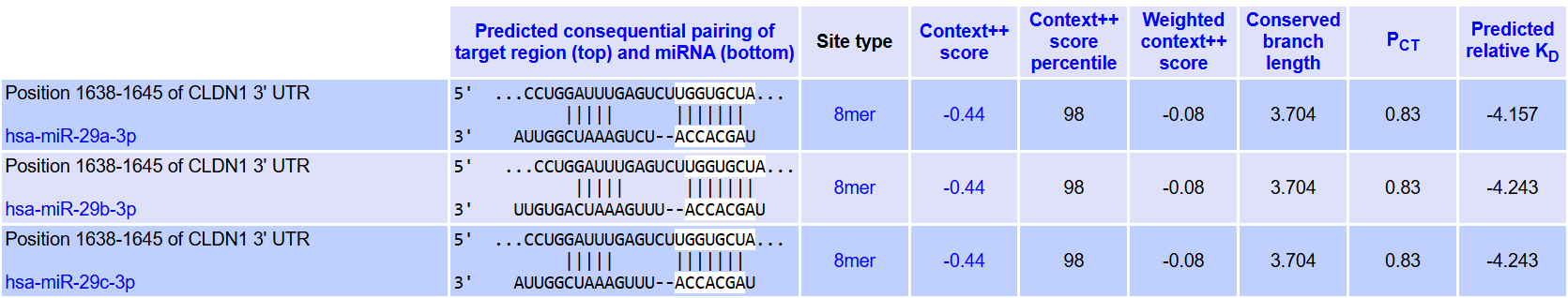 |
| Prediction was performed using TargetScanHuman 8.0 database. Sequence alignments demonstrating the deep evolutionary conservation of the binding sites across multiple vertebrate species for the miR-29-3p family (miR-29a-3p, miR-29b-3p, and miR-29c-3p. Predicted sequence interactions illustrating the specific base pairing between miR-29-3p family and the seed-matching regions within the 3′ UTRs of the target genes. Context++ score, prediction of miRNA targeting efficacy; Conserved branch length, measure of site conservation across 84 vertebrate species; P_CT_, probability of site conservation due to miRNA targeting; Predicted relative K_D_ , binding affinity between the Argonaute–miRNA complex and its target site. | | |
